# Microdomain Protein Nce102 Is a Local Sensor of Plasma Membrane Sphingolipid Balance

**DOI:** 10.1128/spectrum.01961-22

**Published:** 2022-06-27

**Authors:** Jakub Zahumenský, Caroline Mota Fernandes, Petra Veselá, Maurizio Del Poeta, James B. Konopka, Jan Malínský

**Affiliations:** a Department of Functional Organization of Biomembranes, Institute of Experimental Medicine, Academy of Sciences of the Czech Republic, Prague, Czech Republic; b Department of Microbiology and Immunology, School of Medicine, Stony Brook University, Stony Brook, New York, USA; c Division of Infectious Diseases, School of Medicine, Stony Brook University, Stony Brook, New York, USA; d Veterans Administration Medical Center, Northport, New York, USA; Université Côte d'Azur, CNRS, INSERM

**Keywords:** plasma membrane, microdomain, eisosome, sphingolipid, stress sensor

## Abstract

Sphingolipids are essential building blocks of eukaryotic membranes and important signaling molecules that are regulated tightly in response to environmental and physiological inputs. While their biosynthetic pathway has been well-described, the mechanisms that facilitate the perception of sphingolipid levels at the plasma membrane remain to be uncovered. In Saccharomyces cerevisiae, the Nce102 protein has been proposed to function as a sphingolipid sensor as it changes its plasma membrane distribution in response to sphingolipid biosynthesis inhibition. We show that Nce102 redistributes specifically in regions of increased sphingolipid demand, e.g., membranes of nascent buds. Furthermore, we report that the production of Nce102 increases following sphingolipid biosynthesis inhibition and that Nce102 is internalized when excess sphingolipid precursors are supplied. This finding suggests that the total amount of Nce102 in the plasma membrane is a measure of the current need for sphingolipids, whereas its local distribution marks sites of high sphingolipid demand. The physiological role of Nce102 in the regulation of sphingolipid synthesis is demonstrated by mass spectrometry analysis showing reduced levels of hydroxylated complex sphingolipids in response to heat stress in the *nce102*Δ deletion mutant. We also demonstrate that Nce102 behaves analogously in the widespread human fungal pathogen Candida albicans, suggesting a conserved principle of local sphingolipid control across species.

**IMPORTANCE** Microorganisms are challenged constantly by their rapidly changing environment. To survive, they have developed diverse mechanisms to quickly perceive stressful situations and adapt to them appropriately. The primary site of both stress sensing and adaptation is the plasma membrane. We identified the yeast protein Nce102 as a marker of local sphingolipid levels and fluidity in the plasma membrane. Nce102 is an important structural and functional component of the membrane compartment Can1 (MCC), a plasma membrane microdomain stabilized by a large cytosolic hemitubular protein scaffold, the eisosome. The MCC/eisosomes are widely conserved among fungi and unicellular algae. To determine if Nce102 carries out similar functions in other organisms, we analyzed the human fungal pathogen Candida albicans and found that Nce102 responds to sphingolipid levels also in this organism, which has potential applications for the development of novel therapeutic approaches. The presented study represents a valuable model for how organisms regulate plasma membrane sphingolipids.

## INTRODUCTION

Cellular membranes are segmented laterally into functionally distinct microdomains with specific composition and morphology. One of the best-characterized microdomains in the plasma membrane (PM) of eukaryotes is the membrane compartment of Can1 (MCC), discovered in Saccharomyces cerevisiae (1). The MCC accumulates a range of integral proteins, including nutrient importers and tetraspan proteins of the Sur7 and Nce102 families (2–8). It is believed to be rich in ergosterol based on staining with filipin, a fluorescent dye binding sterols (4). The MCC has a furrow shape (9) that is stabilized on the cytosolic side of the plasma membrane by the eisosome, a large protein scaffold made of *Bin/Amphiphysin/Rvs* (BAR) domain-containing proteins Pil1 and Lsp1 that directly bind phosphatidylinositol 4,5-bisphosphate in the plasma membrane and induce membrane curvature (9–16).

The integral plasma membrane protein Nce102 is a structurally and functionally important component of the MCC/eisosome. Its presence in the MCC is essential for the accumulation of nutrient transporters in the microdomain (5) and for the stability of the MCC/eisosome structure as a whole. Deletion of *NCE102* leads to a significant decrease in the number of MCC/eisosome microdomains in the plasma membrane of S. cerevisiae (~50%) (6, 9) and Saccharomyces pombe (*fhn1*Δ, the sole *NCE102* homolog in S. pombe; ~15%) (15) and in the germling heads of Aspergillus nidulans (~30%) (17). The MCC domains formed in the *nce102*Δ mutant lack the typical furrow shape and are flat (9). Interestingly, deletion of as few as 6 amino acid residues from the C terminus of Nce102 is sufficient to abolish Nce102 accumulation in the MCC, resulting in a phenotype comparable with deletion of the whole gene with respect to the sequestration of nutrient transporters in the microdomain as well as its morphology (18).

While the physiological function of Nce102 is still unresolved, it has been shown previously in S. cerevisiae that the protein leaves the MCC following inhibition of sphingolipid biosynthesis in a growing culture. Based on this result, the authors suggested that Nce102 might function as a sphingolipid sensor (6). Sphingolipids, especially inositolphosphoceramides and their mannosylated derivatives, are very abundant in fungal membranes (19, 20), representing the majority of sugar-containing lipids in the plasma membrane of S. cerevisiae (21). Besides their essential structural function, sphingolipids and their precursors (ceramides and long-chain bases [LCBs] dihydrosphingosine and phytosphingosine) are important signaling molecules in a wide range of cellular functions, including response to environmental and endoplasmic reticulum (ER) stress and apoptosis (22–26). Their levels therefore need to be regulated tightly. The current model is that the localization of Nce102 within the plasma membrane responds to the level of complex sphingolipids. Migration of Nce102 out of the MCC in response to the inhibition of sphingolipid biosynthesis results in hyperphosphorylation of the core eisosome component Pil1 and in turn disassembly of the eisosome structure (6). This process leads to the release of Slm1,2 proteins from the MCC/eisosome that activate the target of rapamycin complex 2 (TORC2) (27–29), an important lipid biosynthesis regulator (reviewed in reference 30). While considerable attention has been devoted to the steps leading to the activation of TORC2, and in turn sphingolipid biosynthesis, the function of Nce102 leaving the MCC upon sphingolipid depletion remains unresolved.

Here, we report that the redistribution of Nce102 out of the MCC is a marker of locally increased sphingolipid demand. We show that following the inhibition of sphingolipid biosynthesis, active progression through the cell cycle is necessary for plasma membrane sphingolipid levels to decrease and propose that budding of new cells is the actual mechanism responsible for sphingolipid decrease in the mother cells. In addition, we show an increase in the level of Nce102 in response to elevated sphingolipid demand and internalization of Nce102 to the vacuolar membrane when excess sphingolipid precursors are supplied. Consistently, the *nce102*Δ deletion strain has decreased levels of hydroxylated complex sphingolipids relative to the isogenic wild type in response to heat stress. We further report that Nce102 of the prominent human fungal pathogen Candida albicans behaves similarly to its S. cerevisiae ortholog.

## RESULTS

### The amount of Nce102 in the plasma membrane reflects the current sphingolipid demand.

**FIG 1:**
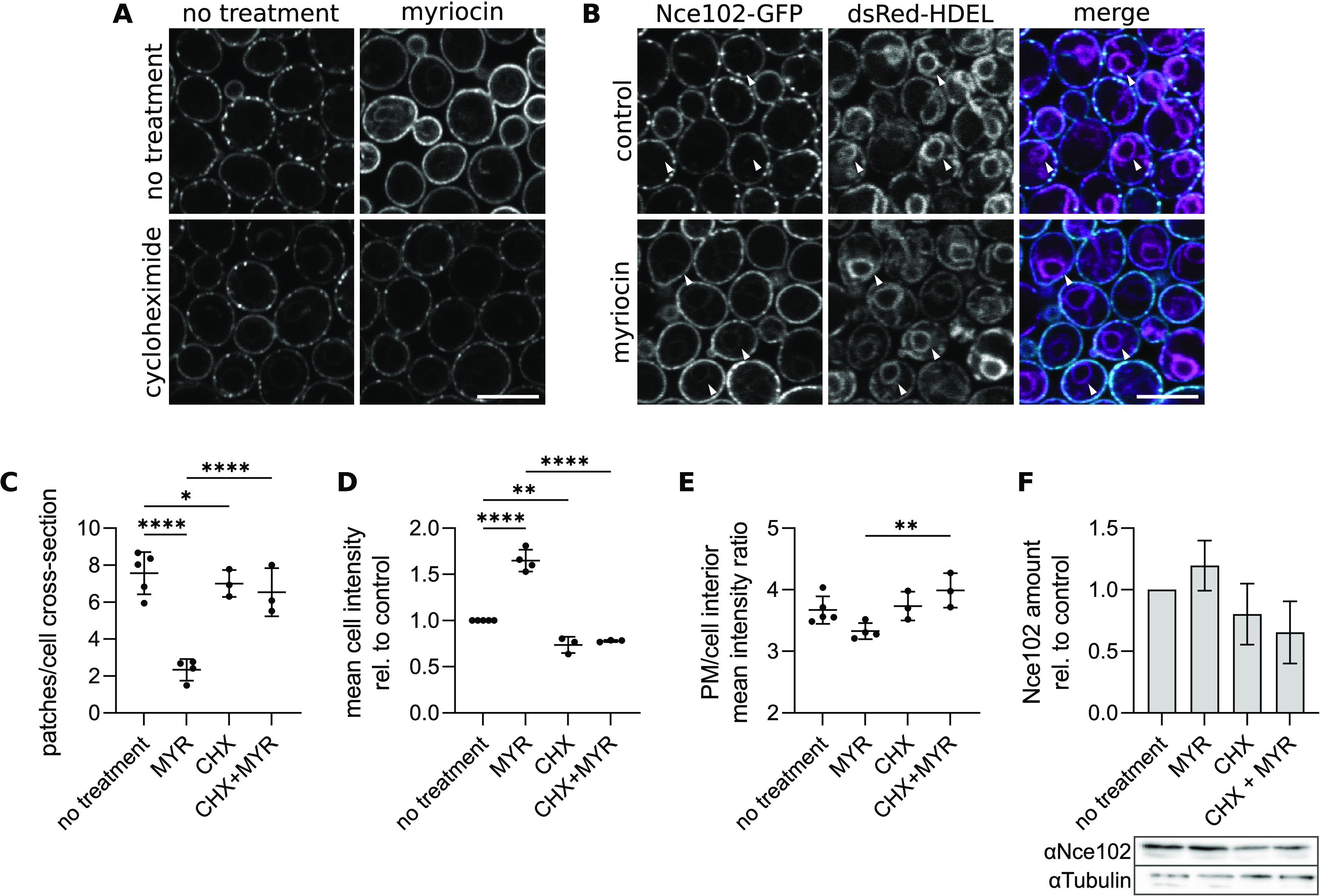
Inhibition of sphingolipid biosynthesis induces the production of Nce102. (A and B) S. cerevisiae cells expressing *NCE102-GFP* either alone (A) or together with the endoplasmic reticulum marker dsRed-*HDEL* (B) cultivated for 6 h and exposed to indicated chemicals for 2 h. Arrowheads in B indicate endoplasmic reticulum. Scale bars: 5 μm. (C to E) Quantification of the number of Nce102-GFP patches per cell cross-section (C), mean cell GFP intensity (D), and the ratio of mean GFP fluorescence intensity in the plasma membrane (PM) and the cell interior (E) in cultures treated as in A. Mean ± SD from 3 to 5 biological replicates (dots; 170 to 230 cells under each condition). *, *P* ≤ 0.05; **, *P* ≤ 0.01; ****, *P* ≤ 0.0001. One-way ANOVA. (F) Western blot quantification of Nce102 amount in cultures treated as in A. Data are presented as mean ± SD from 3 biological replicates. The results did not reach statistical significance (one-way ANOVA) because of variability between experiments. However, the Nce102 amount was always higher in myriocin-treated cultures than in the control in three independent experiments. Shown is a representative membrane with tubulin as the loading control. Myriocin (MYR), 10 μM; cycloheximide (CHX), 100 μg/mL.

The inhibition of the entire sphingolipid biosynthesis pathway by a 2-h myriocin treatment of growing S. cerevisiae cultures leads to the migration of Nce102 out of the MCC into the surrounding plasma membrane (6) (Fig. 1A and C). Investigating this result further, we noticed that the change in Nce102 localization was accompanied by an ~1.65-fold increase in mean cellular Nce102-GFP fluorescence intensity (Fig. 1A and D). The intensity increased more in the cell interior than that in the plasma membrane (Fig. 1E). In addition, GFP-stained intracellular structures emerged that we identified as the endoplasmic reticulum by colocalization with the genetically encoded dsRed-*HDEL* marker (Fig. 1B). These results suggested that inhibition of sphingolipid synthesis triggered *de novo* synthesis of Nce102, which we confirmed by Western blotting after myriocin treatment (Fig. 1F). Consistently, when we simultaneously blocked *de novo* protein synthesis with cycloheximide and sphingolipid biosynthesis with myriocin, the myriocin effect on Nce102 localization and amount was abolished completely (Fig. 1).

**FIG 2:**
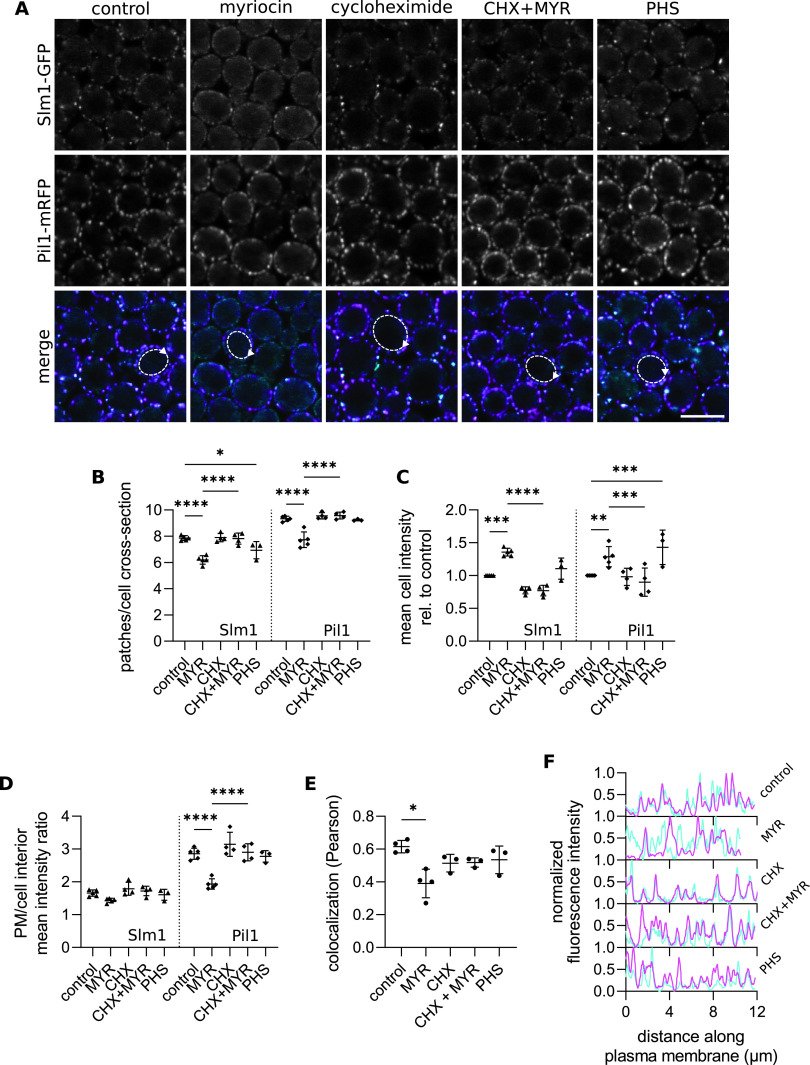
Inhibition of sphingolipid biosynthesis induces the release of Slm1 from the eisosome. (A) Confocal microscopy images of S. cerevisiae cells expressing *SLM1-GFP* together with the core eisosome protein *PIL1*-mRFP cultivated for 6 h and exposed to indicated chemicals for 2 h. Scale bars: 5 μm. Dashed lines with arrows indicate cells and the direction of line intensity plots displayed in F. (B to E) Quantification of the number of Slm1-GFP and Pil1-mRFP patches per cell cross-section (B), mean cell GFP/mRFP intensity (C), the ratio of mean GFP/mRFP fluorescence intensity in the plasma membrane (PM) and the cell interior (D), and Pearson’s colocalization coefficient of Slm1-GFP and Pil1-mRFP (E) in cultures treated as in A. Mean ± SD from 3 to 5 biological replicates (Slm1-GFP, triangles; Pil1-mRFP, diamonds; colocalization, dots; 150 to 200 cells under each condition). *, *P* ≤ 0.05; **, *P* ≤ 0.01; ***, *P* ≤ 0.001; ****, *P* ≤ 0.0001. One-way ANOVA. (F) Normalized line intensity plots of Slm1-GFP (cyan) and Pil1-mRFP (magenta) along the plasma membrane of cells as indicated in A. Myriocin (MYR), 10 μM; cycloheximide (CHX), 100 μg/mL; phytosphingosine (PHS), 20 μM.

According to the current model, migration of Nce102 out of the MCC destabilizes the eisosome leading to the release of Slm1,2 proteins from the MCC/microdomain and in turn activation of TORC2 and sphingolipid biosynthesis (28, 31, 32). To verify this process, we monitored changes in Pil1-mRFP and Slm1-GFP localization following treatment of cells with myriocin. We found a decrease in the number of plasma membrane patches of both Pil1 and Slm1 and a relative increase in intracellular intensity, pointing to a decreased interaction of both proteins with the plasma membrane (Fig. 2B and C) (shown previously for Pil1 [10]). The overlap of Slm1-containing membrane patches with the Pil1-mRFP signal decreased significantly following myriocin treatment (Fig. 2A, E, and F; see Fig. S1 in the supplemental material [tangential planes]), consistent with previously published results (8, 28). Analogous to Nce102, the mean cellular intensity of Slm1-GFP and Pil1-mRFP increased following the inhibition of sphingolipid biosynthesis (Fig. 2C). When we simultaneously blocked the *de novo* protein synthesis by cycloheximide, Slm1-GFP retained its colocalization with Pil1-mRFP, and changes in intensity and distribution were abolished for both proteins (Fig. 2; Fig. S1, tangential planes). While our results show that Pil1 and Slm1 behave analogously to Nce102 in response to the inhibition of the entire sphingolipid biosynthesis pathway, they also show that the reported changes are significantly more extensive in the case of Nce102 (compare Fig. 1 and 2). We also found that Sur7, another integral MCC protein, responds only marginally to myriocin treatment (see Fig. S2A to C in the supplemental material). These results highlight the prominence of Nce102 in the studied processes.

**FIG 3:**
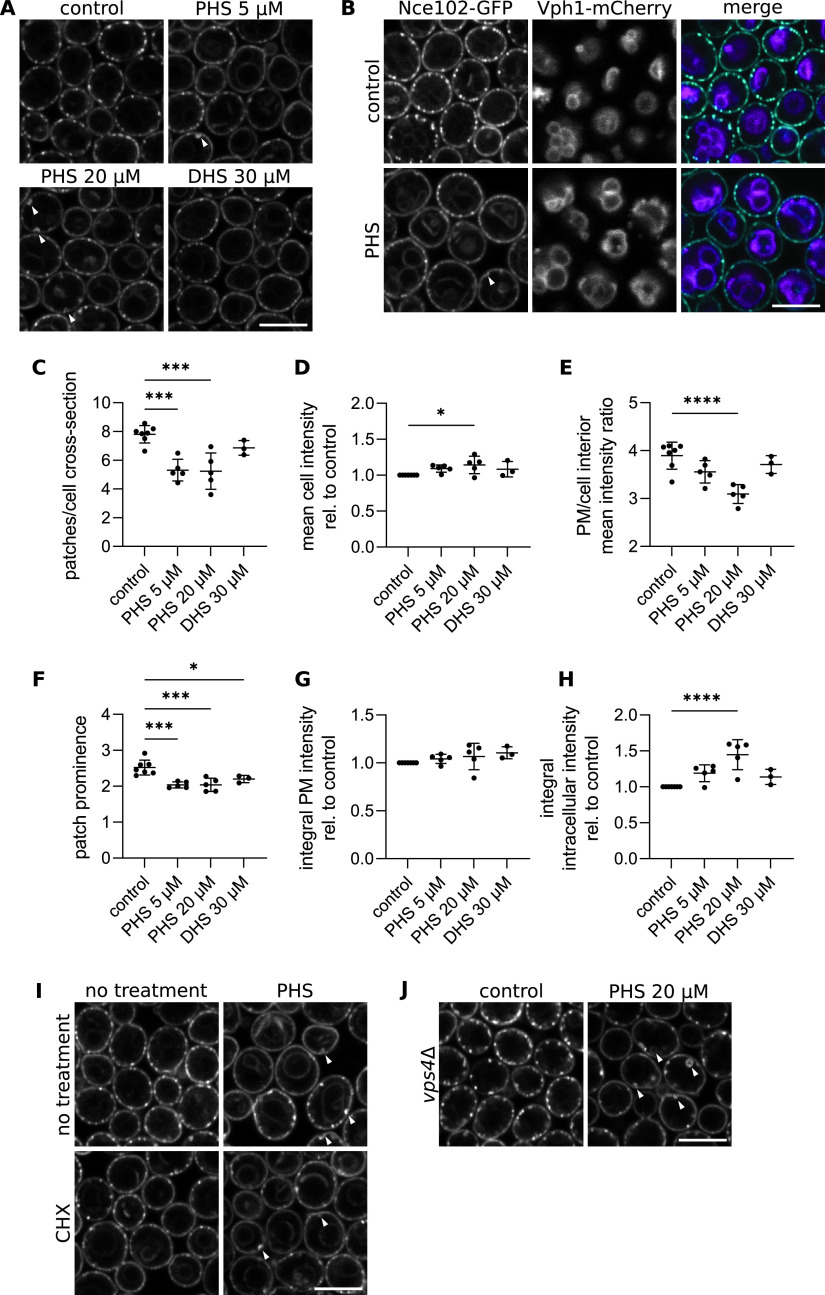
Nce102 migrates out of the MCC and is internalized in the presence of excess sphingolipid precursors. (A and B) Confocal microscopy images of S. cerevisiae cells expressing *NCE102-GFP* either alone (A) or together with the vacuole membrane marker Vph1-mCherry (B) cultivated for 6 h and exposed to indicated chemicals for 2 h. Scale bars: 5 μm. Arrowheads in A indicate plasma membrane invaginations. (C to H) Quantification of the number of Nce102-GFP patches per cell cross-section (C), mean cell GFP intensity (D), the ratio of mean GFP fluorescence intensity in the plasma membrane (PM) and the cell interior (E), prominence of patches (ratio of intensities in peaks and valleys in line plots along plasma membranes of individual cells) (F), and mean fluorescence intensity in the plasma membrane (G) and cell interior (H) in cultures treated as in A. Mean ± SD from 3 to 5 biological replicates (dots; 170 to 230 cells under each condition). *, *P* ≤ 0.05; ***, *P* ≤ 0.001; ****, *P* ≤ 0.0001. One-way ANOVA. (I and J) Confocal microscopy images of S. cerevisiae cells expressing *NCE102-GFP* either in the wild type (I) or in a *vps4* deletion mutant (J) cultivated for 6 h and exposed to indicated chemicals for 2 h. Arrowheads indicate plasma membrane invaginations and intracellular vesicles. Scale bars: 5 μm. Cycloheximide (CHX), 100 μg/mL; dihydrosphingosine (DHS), concentration as indicated; phytosphingosine (PHS), concentrations in B and I as indicated in (A), 20 μM in (J).

Since Nce102 leaves the MCC in response to sphingolipid insufficiency, we also investigated its behavior under conditions of elevated sphingolipid content. To achieve this inquiry, we supplemented the culture medium with dihydrosphingosine (DHS) or phytosphingosine (PHS), long-chain bases (LCBs) used by the cells as sphingolipid precursors. The ability of yeast cells to utilize exogenous LCBs for the biosynthesis of ceramide and complex sphingolipids has been documented previously (6, 27, 33–39). A 2-h treatment of exponentially growing cells with either PHS or DHS resulted in a decrease in the number and prominence (i.e., the ratio of fluorescence intensity in patches and surrounding plasma membrane) of Nce102-GFP patches, indicating their partial disintegration (Fig. 3A, C, and F; see Fig. S3 in the supplemental material, tangential planes). The mean plasma membrane intensity was not affected by the addition of exogenous LCBs, and the cellular intensity increased slightly in response to 20 μM PHS. On the other hand, invaginations in the plasma membrane appeared and the mean intracellular intensity increased significantly, resulting in a shift in the distribution of the signal in favor of the intracellular space (Fig. 3D, E, G, and H). Consistently, intracellular structures stained with GFP emerged, which we identified as vacuoles by colocalization with Vph1-mCherry, a subunit of the vacuolar V-ATPase (Fig. 3B). These results indicate an internalization of Nce102 from the plasma membrane to the membrane of the vacuole in the excess of sphingolipids. To clearly identify the origin of the vacuolar Nce102, we inhibited *de novo* protein synthesis by cycloheximide prior to PHS addition. Neither the migration of Nce102-GFP out of the MCC nor its targeting to the vacuolar membrane were affected (Fig. 3I), indicating that the existing protein is redistributed. Furthermore, we have shown previously that as the cell culture ages and overall sphingolipid content of the cell increases, a subpopulation of Nce102 migrates from the plasma membrane to the membrane of the vacuole via the multivesicular body pathway (40). Consistently, in cells lacking Vps4, which have significantly decreased endocytosis and defects in late endosome-to-vacuole sorting (41), Nce102-GFP migrated out of the MCC in response to PHS addition, but its targeting to the vacuolar membrane was abolished. In addition, the incidence of PHS-triggered plasma membrane invaginations increased compared with that of the wild type (Fig. 3J). Our results hence indicate that in the presence of surplus sphingolipid precursors, Nce102-GFP redistributes from the MCC into the surrounding plasma membrane and is in turn internalized to the vacuolar membrane.

In contrast to the effect on Nce102 distribution, the PHS addition caused only small changes in the number of Pil1 and Slm1 patches, while leading to an increase in the mean cellular intensity, more extensive in the plasma membrane than in the cell interior (Fig. 2A to D), and an increase in patch fluorescence intensity (Fig. S1). The colocalization of the two proteins was unaffected by the addition of exogenous LCBs (Fig. 2A, E, and F). In addition, we found analogous changes in the distribution of Lsp1, a Pil1 homolog, in response to both PHS addition and myriocin treatment (Fig. S2D to G). These results indicate that in contrast to Nce102, Pil1 and Slm1 tend to increase their accumulation in the MCC/eisosome when there is an excess of sphingolipid synthesis precursors.

### Nce102 migration out of the MCC in response to sphingolipid inhibition requires active budding.

We examined in more detail the necessity of active *de novo* protein synthesis for the migration of Nce102 out of the MCC in response to sphingolipid biosynthesis inhibition. The cycloheximide-induced block of protein synthesis leads ultimately to cell cycle arrest (42). As the cell cycle progression is tightly linked to the sphingolipid levels (43, 44), we hypothesized that the migration of Nce102 out of the MCC upon myriocin treatment required progression through the cell cycle.

**FIG 4:**
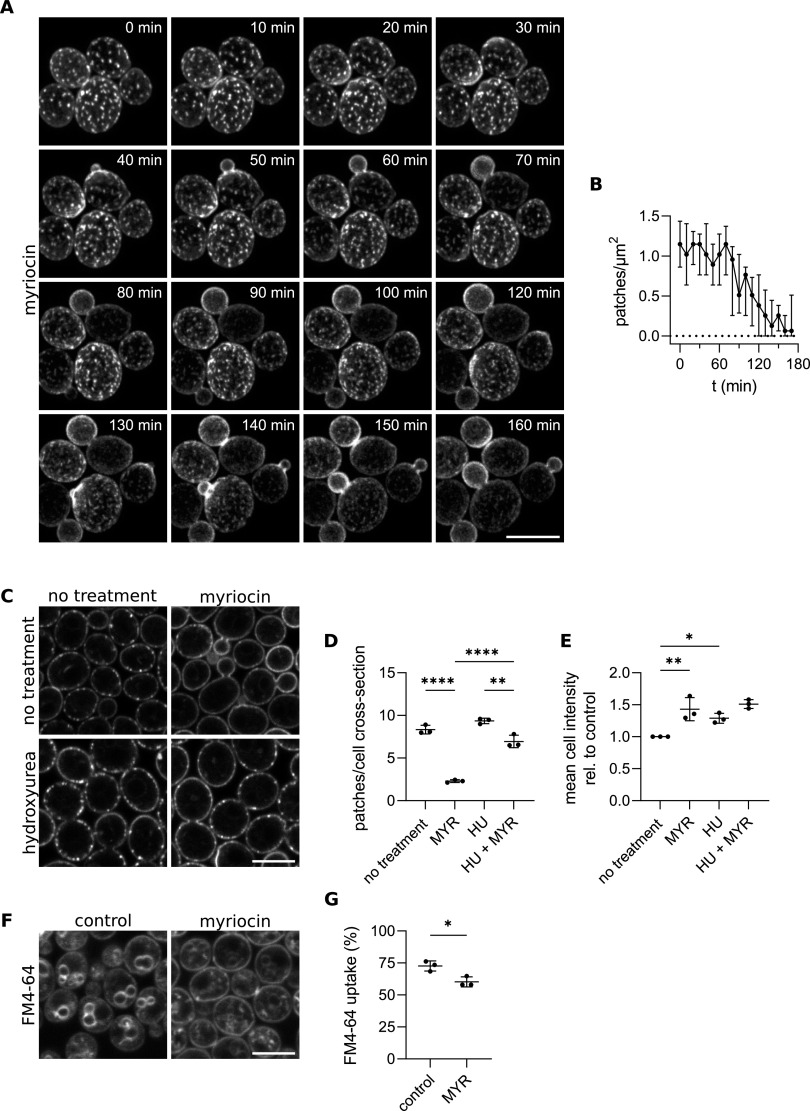
Nce102 migration out of the MCC in response to sphingolipid inhibition is dependent on active budding. (A) Confocal microscopy images of S. cerevisiae cells expressing *NCE102-GFP* cultivated for 6 h, treated with myriocin at a time (*t*) of 0 min, and imaged in a time-lapse manner. Maximum intensity projections of 9 subsequent focal planes are presented. (B) The dependence of plasma membrane Nce102-GFP patch density on time quantified in images from a single time-lapse experiment (representative cropped area in A). Data are presented as median and interquartile range from 25 cells in each image. (C) Confocal microscopy images of S. cerevisiae cells expressing *NCE102-GFP* cultivated for 5 h, treated with hydroxyurea for 3 h, and subsequently treated for 2 h with myriocin. (D and E) Quantification of the number of Nce102-GFP patches per cell cross-section (D) and mean cell intensity (E) in cultures treated as in C. Mean ± SD from 3 biological replicates (dots; 100 to 150 cells per condition). *, *P* ≤ 0.05; **, *P* ≤ 0.01; ****, *P* ≤ 0.0001. One-way ANOVA. (F) Confocal microscopy images of S. cerevisiae cells cultivated for 6 h, stained with FM4-64 dye, and treated as indicated, followed by a 2-h cultivation. (G) Quantification of FM4-64 uptake by the cells treated as in F, calculated as the ratio of integral GFP intensity in the cell interior and in the whole cell. Mean ± SD from 3 biological replicates (dots; 150 to 230 cells per condition). *, *P* = 0.0161. Paired *t* test. Myriocin (MYR), 10 μM; hydroxyurea (HU), 200 mM; FM4-64 (*N*-(3-triethylammoniumpropyl)-4-(6-(4-(diethylamino) phenyl) hexatrienyl) pyridinium dibromide), 2 μM. In A, C, and F, scale bars: 5 μm. In D, E, and G, mean ± SD from 3 biological replicates (150 to 220 cells under each condition; dots).

First, we considered that myriocin is not growth inhibitory within the time frame of our experiments (45). Furthermore, in our time-lapse experiments, it took ~80 min for the Nce102-GFP patch density to drop to one-half (Fig. 4A and B; see Movie S1 in the supplemental material), which was sufficient for cells with small buds at *t* = 0 min to finish cytokinesis and for newly formed buds to grow almost to full size. As the buds increased in size, the patchy pattern vanished gradually in their respective mother cells, while Nce102-labeled MCC patches were not formed at all in the growing daughters (Fig. 4A; Movie S1). Cells that did not bud within the time frame of the experiment (120 min) retained the patchy Nce102-GFP distribution in the plasma membrane.

To test directly whether an active cell cycle is required for the myriocin-induced change in Nce102 localization and abundance, we treated exponentially growing cells with 200 mM hydroxyurea for 3 h before inhibiting sphingolipid biosynthesis by 2-h exposure to myriocin. At this concentration, hydroxyurea completely inhibits DNA synthesis resulting in cell cycle arrest in the S-phase, while decreasing *de novo* protein synthesis by only ~30% (46, 47). As apparent from Fig. 4C to E, arresting the cell cycle before myriocin addition prevented changes in both the localization and abundance of Nce102. Based on our results, we conclude that cell cycle progression is required for Nce102 migration out of the MCC in response to inhibition of sphingolipid biosynthesis.

A decrease of complex sphingolipid content in response to myriocin treatment has been reported previously (39, 48–50). Curiously, even though sphingolipid biosynthesis is blocked, the cells continue to bud and grow normally for up to ~5 h (45), which suggests that pre-existing sphingolipids are being used in the newly synthesized bud membranes. We propose that following myriocin treatment, sphingolipids necessary for the bud plasma membrane construction are rerouted to the growing bud from the mother cell, leading to a decrease of complex sphingolipid levels in its plasma membrane. This process triggers the migration of Nce102 out of the MCC. As the septum at the bud neck constitutes a strong diffusion barrier (51), the necessary sphingolipids need to be internalized from the plasma membrane of the mother cell and passed to the progeny via organelle inheritance. Therefore, we monitored the uptake of a known endocytosis reporter, FM4-64, in mother cells following myriocin treatment and found that while it was decreased significantly by the sphingolipid biosynthesis inhibition (endocytosis requires an abundance of long-chain bases [36, 52]), 60% of the dye was still internalized (Fig. 4F and G). Lowering of sphingolipid levels in the plasma membrane of the mother cell by endocytosis is hence plausible.

### Acute inhibition of ergosterol biosynthesis does not affect Nce102 localization.

**FIG 5:**
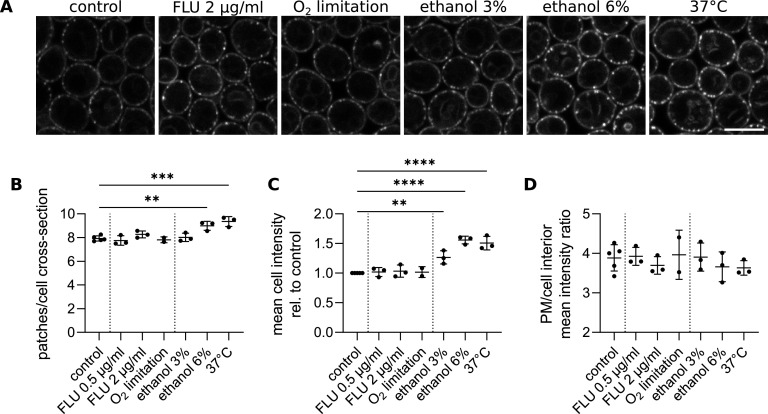
Nce102 localization and abundance change in response to increasing plasma membrane fluidity and sphingolipid levels. (A) Confocal microscopy images of S. cerevisiae cells expressing *NCE102-GFP* cultivated for 6 h and exposed to indicated stress conditions for 2 h. Scale bar: 5 μm. (B to D) Quantification of the number of Nce102-GFP patches per cell cross-section (B), mean cell intensity (C), ratio of mean GFP fluorescence intensity in the plasma membrane (PM) and the cell interior (D) in cultures treated as in A. Mean ± SD from 3 to 5 biological replicates (dots; 300 to 400 cells under each condition). *, *P* ≤ 0.05; **, *P* ≤ 0.01; ***, *P* ≤ 0.001; ****, *P* ≤ 0.0001. One-way ANOVA. No statistical difference was found between conditions in D. Vertical dotted lines separate groups of stress conditions, left to right, as follows: control, ergosterol biosynthesis inhibition, and increase of membrane fluidity. FLU, fluconazole.

Preferential localization of Nce102 into ergosterol-enriched domains of cellular membranes (4, 40) suggests that a decrease in ergosterol level could result in changes in the protein distribution. To test this, we inhibited the ergosterol biosynthesis pathway by the addition of fluconazole. It was reported previously that S. cerevisiae cells cultivated in the presence of 1.5 μg/mL fluconazole for 24 h have reduced ergosterol levels by 80% (53). Exposure of exponentially growing S. cerevisiae cells to 2 μg/mL fluconazole for 2 h caused no significant change in either localization or mean cellular fluorescence of Nce102-GFP (Fig. 5; Fig. S3, tangential planes).

Ergosterol synthesis can also be inhibited by limiting the supply of oxygen, which is required for the production of heme, an ergosterol precursor (54). S. cerevisiae cultures grown exponentially in hypoxic conditions produce only ~10% ergosterol of the amount made by their aerated counterparts (55). In addition, a lack of oxygen causes a decrease in plasma membrane fluidity due to the defective desaturation of fatty acids (56, 57). As see after fluconazole treatment, we found no changes in Nce102 localization and abundance in cells exposed to oxygen-limiting conditions for 2 h (Fig. 5; Fig. S3, tangential planes). We conclude that Nce102 distribution is not sensitive to acute changes in ergosterol levels.

### Acute increase in plasma membrane fluidity induces the production of Nce102.

To increase the membrane fluidity of exponentially growing cells, we either shifted the culture from optimal 28 to 37°C or added ethanol to the growth medium (58–61). The increase in membrane fluidity is normally compensated by activating the biosynthesis of the complex sphingolipids that increase rigidity (62–65). Upon transferring 28°C-grown exponential culture to 37°C for 2 h, the number of plasma membrane Nce102 patches increased by ~10% (Fig. 5A and B) and the mean cellular Nce102-GFP intensity increased ~1.5-fold (to the same degree in the plasma membrane and cell interior; Fig. 5; Fig. S3, tangential planes), indicating an increase in Nce102 protein level. Exposing exponential phase cells to ethanol, another membrane fluidity-increasing agent, caused an increase in both the number of plasma membrane Nce102 patches and the mean cellular fluorescence. These effects increased with ethanol concentration and at 6% were comparable with the shift to 37°C (Fig. 5; Fig. S3A to E, tangential planes). To exclude nonspecific effects of elevated temperature and ethanol on GFP fluorescence, we monitored Sur7-GFP under the same conditions. The fluorescence intensity of Sur7-GFP decreased when cells were cultivated at 37°C and was not affected by ethanol or other stress conditions (see Fig. S4F in the supplemental material). Therefore, we conclude that acutely increasing the fluidity of the plasma membrane triggers the synthesis of Nce102 and its targeting into the MCC.

### Nce102 localization and abundance changes following chronic stress.

**FIG 6:**
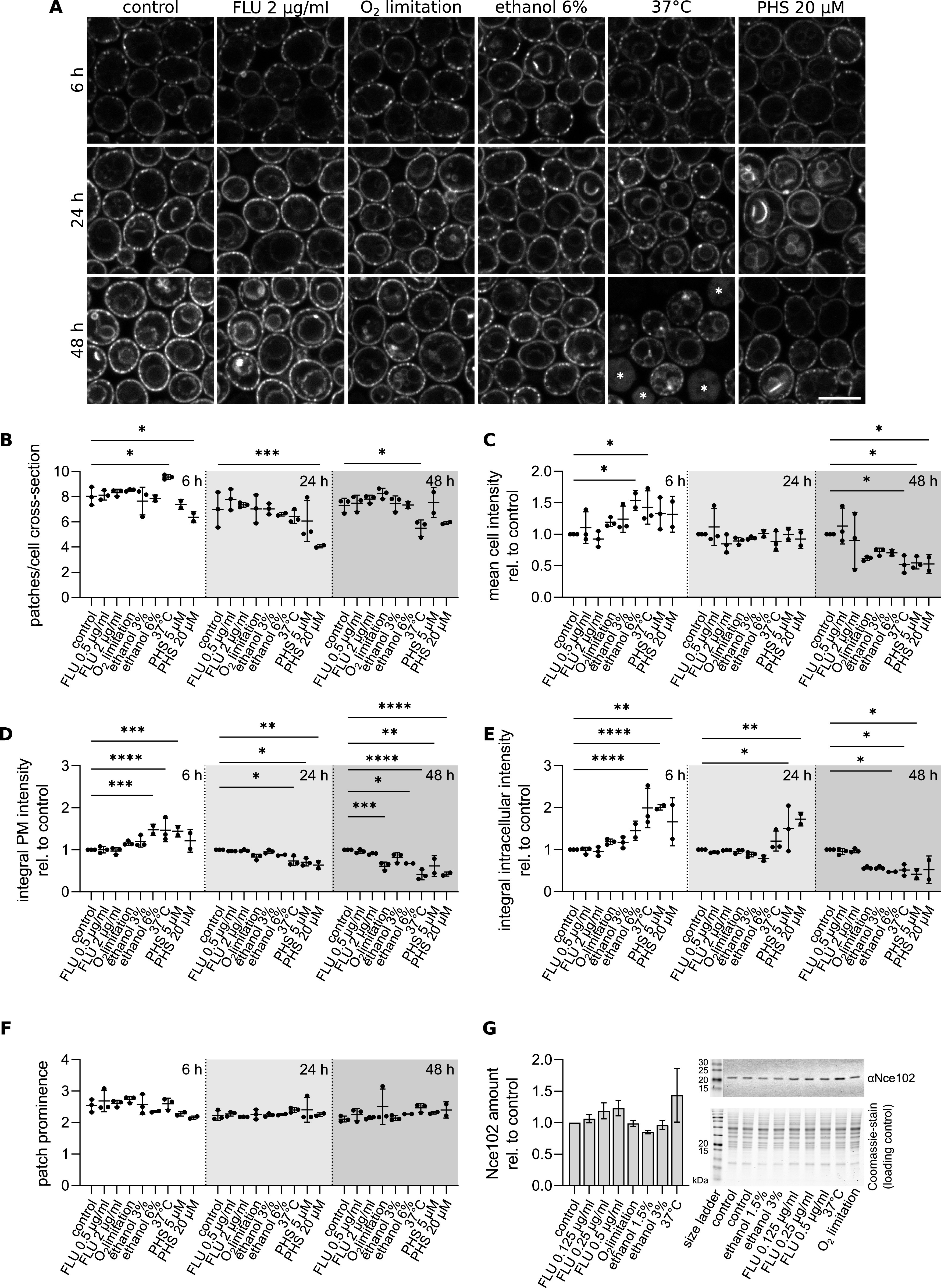
Nce102 response to chronic stress. (A) Confocal microscopy images of S. cerevisiae cells expressing *NCE102-GFP* treated with indicated stress upon inoculation and cultivated for the indicated time. Scale bars: 5 μm. White asterisks indicate dead cells. (B to F) Quantification of the number of Nce102-GFP patches per cell cross-section (B), mean cell GFP intensity (C), integral intensity in the plasma membrane (PM) (D) and in the intracellular space (E), and patch prominence, i.e., GFP intensity in the patches relative to the surrounding plasma membrane, (F) in cell cultures treated as in A. Mean ± SD from 3 biological replicates (dots; 150 cells in 48 h 37°C condition, dead cells were excluded from analysis; 250 to 400 cells in all other conditions). *, *P* ≤ 0.05; **, *P* ≤ 0.01; ***, *P* ≤ 0.001; ****, *P* ≤ 0.0001. One-way ANOVA. Vertical dotted lines separate cultivation times. (G) Western blot quantification of Nce102 amount in S. cerevisiae cultures grown for 48 h under chronic exposure to indicated stress. Data are presented as mean ± SD from 3 biological replicates. Because of the variability between experiments, no statistically significant difference between the treatments was found (one-way ANOVA). However, the reported trend was comparable in three independent experiments. Representative Western blot membrane and Coomassie-stained gel were used as the loading control. Note the different order of treatments in the graph and on the gel/membrane. FLU, fluconazole; PHS, phytosphingosine.

As we found that Nce102 plays a sensor role in the regulation of cellular response to acute sphingolipid imbalance and increased plasma membrane fluidity, we investigated if the protein is involved also in long-term adaptation to stress. Therefore, we applied the stress conditions described above at culture inoculation and monitored the behavior of Nce102 over time. Cell cultures grown for 6 h after the addition of surplus ethanol and sphingolipid precursors, as well as under elevated temperature, exhibited characteristics comparable to exponential cells exposed to these stresses acutely for 2 h (Fig. 6A to F; Fig. S4). Specifically, mean cellular GFP fluorescence increased, while GFP-stained intracellular structures became more prominent, especially following PHS addition. The excess of sphingolipid precursors also caused a partial loss of Nce102 patches. Inhibition of ergosterol synthesis by either fluconazole or limiting oxygen supply had again no significant effect on Nce102.

Cultivation of nonstressed cells for 24 and 48 h resulted in a gradual increase of their overall Nce102-GFP fluorescence, as well as pronounced vacuolar localization (Fig. 6A), consistent with our previous study (40). Cells grown either at an elevated temperature (37°C) or supplemented with exogenous sphingolipid precursors for 24 h exhibited a significant increase in the intracellular GFP fluorescence relative to the control, while the intensity in the plasma membrane decreased (Fig. 6D and E). In the case of PHS supplementation, this finding was accompanied by a decrease in the number of Nce102-GFP patches in the plasma membrane (Fig. 6A and B). However, the prominence of the remaining patches did not change (Fig. 6F), suggesting that some microdomains disassemble completely while others remain intact. Exposing the cultures to either heat stress or excess PHS for 48 h reduced the mean cellular Nce102-GFP fluorescence, similarly in the plasma membrane and the cytosol (Fig. 6A and C to E). The cultures cultivated in the presence of fluconazole, ethanol, and under conditions of limited oxygen supply for 24 to 48 h exhibited characteristics comparable to the control cells, suggesting possible adaptation of the growing cultures. According to a biochemical analysis of S. cerevisiae cultures, a 48-h exposure of cultures to studied stress conditions resulted in an increase in cellular Nce102 protein amount in response to fluconazole and increased temperature (Fig. 6E; see negative control in Fig. S5 in the supplemental material). We conclude that Nce102 is more sensitive to acute disruption of sphingolipid balance and increasing plasma membrane fluidity than to changes induced by long-term adaptation to stress.

### Nce102 fine-tunes sphingolipid synthesis in response to chronic stress.

We show above that the cellular localization and amount of Nce102 respond to changes in the sphingolipid abundance and fluidity of the plasma membrane. This mechanism might be a tool for tuning TORC2 activity and in turn sphingolipid biosynthesis. Specifically, Nce102 leaving the MCC following myriocin treatment destabilizes the eisosome, releasing Slm1,2 that activate TORC2. In addition, Nce102 itself has been proposed to interact directly with TORC2, enhancing its activation (66). Removing Nce102 from the plasma membrane (as after PHS addition) would therefore prevent excessive TORC2 activation. To verify Nce102 involvement in the regulation of sphingolipid synthesis, we analyzed changes in cellular sphingolipid composition in response to the studied stress conditions using mass spectrometry and assessed the contribution of the Nce102 protein to these changes.

**FIG 7:**
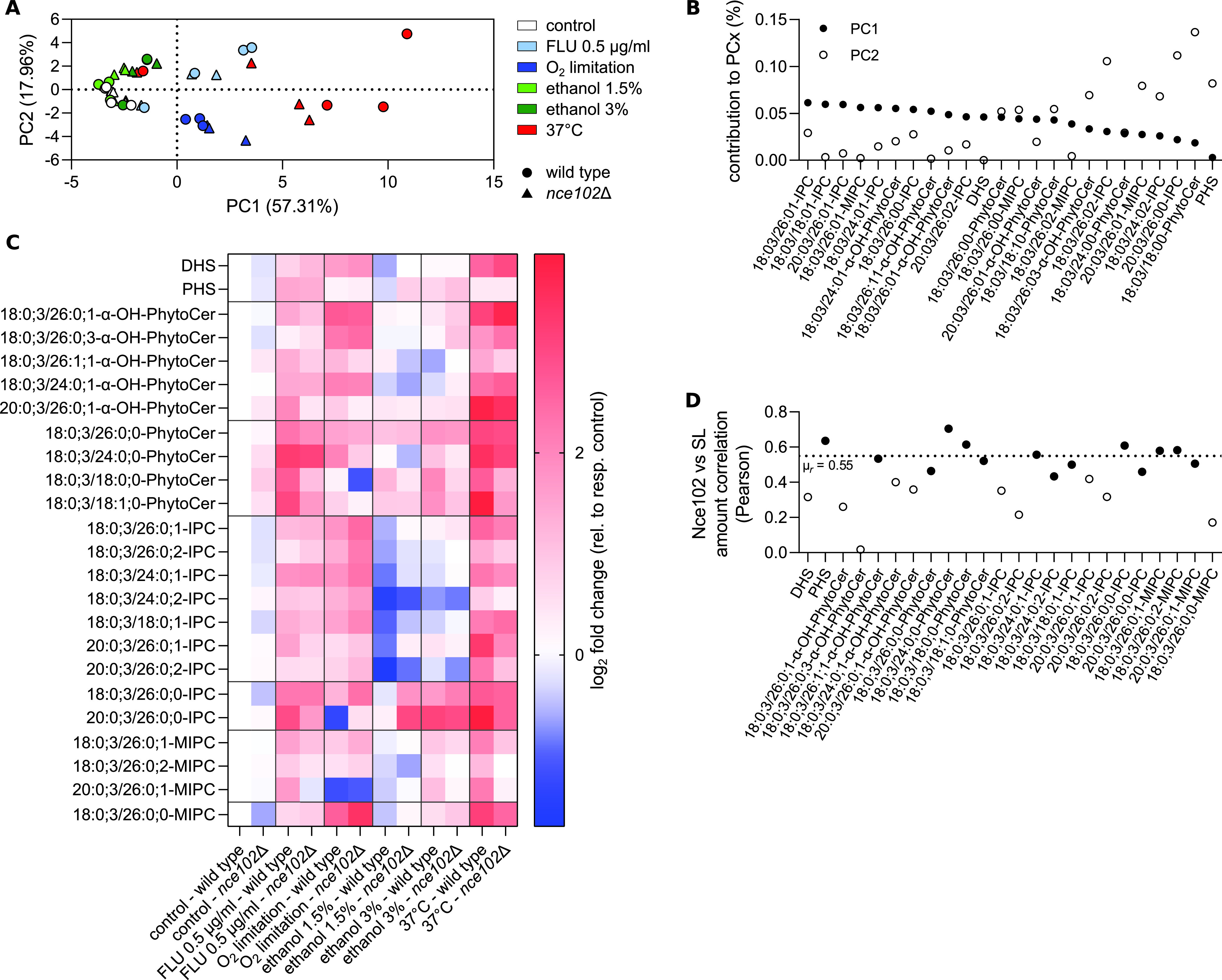
Mass spectrometry analysis of sphingolipid content. S. cerevisiae wild type and *nce102*Δ cultures were treated with indicated stress conditions upon inoculation and cultivated for 48 h (same as in Fig. 6); 3 to 4 biological replicates. (A) Principal-component analysis for sphingolipid amount across samples. (B) Contribution of individual lipids to PC1 (full circles) and PC2 (full circles). (C) Log_2_ fold changes in sphingolipid amounts in response to indicated chronic stress relative to respective control; *nce102*Δ control relative to wild-type control. Note that mannosyl-di-(inositolphosphoryl)-ceramides [M(IP)_2_Cs] were not detected in our assay. (D) Analysis of the correlation (Pearson’s coefficient) between the amount of Nce102 (as reported in Fig. 6) and individual lipids. Full symbols, statistically significant correlation (two-tailed *P* ≤ 0.05); empty symbols, correlation below threshold of statistical significance (*P* > 0.05); *μ*_r_, mean Pearson’s correlation coefficient calculated from statistically significant values. FLU, fluconazole; LCBs, long-chain bases; Cer, ceramide; PhytoCer, phytoceramide; IPC, inositol phosphoceramides; MIPCs, mannosyl inositolphosphoceramides; SL, sphingolipids; OH-, prefix indicates hydroxylation of respective lipids.

To get an initial insight into the results, we performed principal-component analysis (PCA) that analyzes correlations among the samples and creates a two-dimensional (2D) plot, in which samples with similar sphingolipid profiles cluster together. The PCA showed clear clustering and separation of the cultures exposed to heat stress or when ergosterol biosynthesis was challenged, either by the presence of fluconazole or by growing the cells under oxygen-limiting conditions (Fig. 7A). Ethanol-treated cultures also separated from controls, in a concentration-dependent manner. The highest contributors to the first principal component (PC1; linear combination of individual lipids) are monohydroxylated inositolphosphoceramides (IPCs) and phytoceramides (Fig. 7B), indicating that the biggest differences between the samples are due to changes in these lipid classes. Differences between the *nce102*Δ deletion strain and the wild type were not clear.

An analysis of the individual lipid profiles indicated that, already in the absence of stress, deletion of *NCE102* caused a slight decrease in the overall levels of long-chain bases (LCBs), inositolphosphoceramides (IPCs), and mannosyl inositolphosphoceramides (MIPCs) (Fig. 7C), supporting the proposed role of Nce102 in the activation of sphingolipid biosynthesis. Interestingly, the levels of phytoceramides were higher in the deletion mutant. The stress conditions except 1.5% ethanol triggered a general increase in sphingolipid levels, including long-chain bases (Fig. 7C). The largest increase in sphingolipid levels took place when cells were cultivated at 37°C, pointing to strong activation of *de novo* sphingolipid biosynthesis. While the degree of the stress-induced changes was not significantly different in the *nce102*Δ deletion mutant for most of the individual lipids and stress conditions (see Table S1 and Fig. S6 in the supplemental material), the increase of hydroxylated IPCs and MIPCs as groups in response to cultivation at elevated temperature was significantly affected by the absence of Nce102 (Fig. 7C; see Fig. S7 in the supplemental material). Following cultivation with either ethanol or fluconazole, the nonhydroxylated phytoceramides and IPCs increased more extensively than other sphingolipids. In the case of ethanol, however, there was an overall decrease in hydroxylated IPC species (more extensive at 1.5%) suggesting a more general remodeling of the plasma membrane lipid landscape. The changes, in either direction, were again less extensive in the *nce102*Δ deletion mutant in response to both ethanol and fluconazole. Under conditions of limited oxygen supply, there was an overall increase in the levels of all sphingolipid classes, except for a few specific lipids. While the increase in the amount of phytoceramides was less extensive in the absence of Nce102, all detected inositolphosphoceramides and mannosyl inositolphosphoceramides increased more in the *nce102*Δ mutant in response to oxygen limitation. The reported changes in the level of specific lipids and lipid classes (Fig. 7C; Fig. S6 and S7) in response to various conditions are reminiscent of the changes in the Nce102 protein level. We found a positive correlation between these two parameters (Fig. 7D).

### Nce102 behavior is conserved in the human fungal pathogen C. albicans.

**FIG 8:**
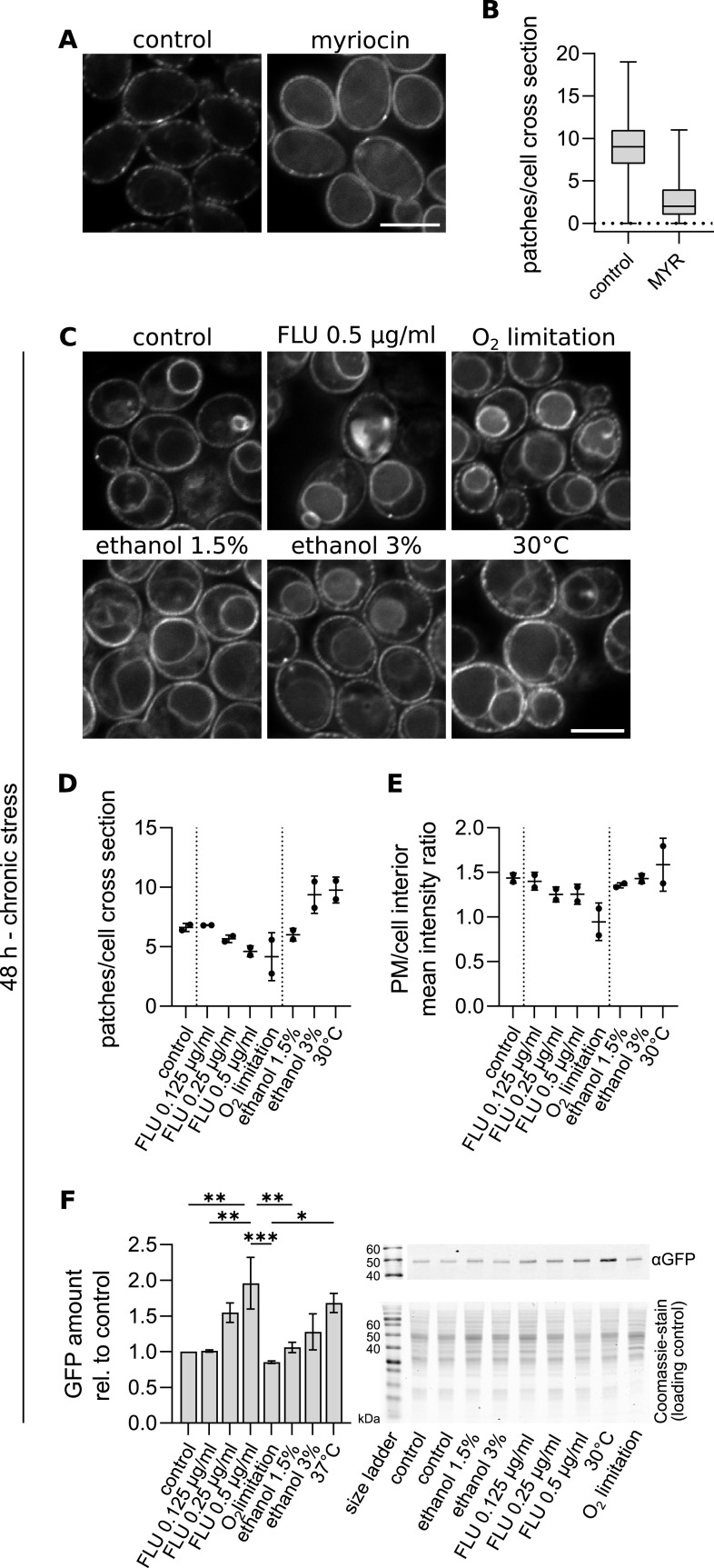
Nce102 behaves analogously in C. albicans and S. cerevisiae. (A and C) Deconvolved wide-field fluorescence microscopy images of C. albicans cells expressing Ca*NCE102-GFP* cultivated for 6 h and exposed to myriocin for 2 h (A) or treated with indicated stress upon inoculation and cultivated for 48 h (C). Scale bars: 5 μm. (B and D) Quantification of the number of *Ca*Nce102-GFP patches per cell cross-section following either 2-h myriocin treatment (B) or chronic exposure to indicated stress (C). (E) Quantification of the ratio of mean GFP fluorescence intensity in the plasma membrane (PM) and the cell interior following chronic exposure to indicated stress. No statistically significant difference between the treatments was found (one-way ANOVA). (F) Western blot quantification of Nce102 amount in C. albicans cultures grown for 48 h under chronic exposure to indicated stress. Mean ± SD from 3 biological replicates. Representative Western blot membrane and Coomassie-stained gel were used as the loading control. Note the different order of treatments in the graph and on the gel/membrane. Myriocin (MYR), 10 μM; FLU, fluconazole. In B, data are represented as box plots with median and range (minimum to maximum) indicated, with one biological replicate (control, 975 cells; myriocin, 512 cells). In D and E, mean values ± SD from 2 biological replicates (≥100 cells under each condition; dots). In D and F, *, *P* ≤ 0.05; **, *P* ≤ 0.01; ***, *P* ≤ 0.001. One-way ANOVA.

As the MCC/eisosome microdomain is evolutionarily conserved throughout the fungal kingdom (9, 67), we tested whether Nce102 exhibits the same behavior in the opportunistic human fungal pathogen Candida albicans. In healthy humans, C. albicans is a harmless inhabitant of the gastrointestinal tract (68). Therefore, cultures of this yeast grown at 37°C were used as controls. Just like in S. cerevisiae, a 2-h myriocin treatment of exponentially growing C. albicans cells leads to migration of Nce102-GFP out of the MCC (Fig. 8A and B), suggesting a conservation of the sensor function of Nce102. However, we did find an interesting difference between these two species in that, while Sur7-GFP of S. cerevisiae did not respond to myriocin (Fig. S2), the C. albicans homolog Sur7 migrated almost completely out of the MCC in response to myriocin treatment (Fig. S5) (69).

C. albicans cultures grown for 48 h at 42°C exhibited large-scale dying of the cells, as did cultures exposed chronically to 6% ethanol and as low as 1 μg/mL fluconazole (Fig. S5). Therefore, we compared *Ca*Nce102-GFP localization and the amount between cultures grown at optimal 37°C and suboptimal 30°C and used lower concentrations of ethanol and fluconazole. Cultivation at 30°C resulted in an increased number of plasma membrane Nce102-GFP patches (Fig. 8C and D), which correlated well with the increased cellular amount of *Ca*Nce102-GFP (Fig. 8F). Inhibition of ergosterol synthesis by either fluconazole addition or limiting oxygen supply resulted in a decrease in the number of Nce102-GFP patches and an increase of GFP intensity inside the vacuole (resulting in a shift of the PM/cell interior GFP intensity ratio toward lower values). Since Nce102 is a transmembrane protein tagged with GFP on its cytosolic C terminus, diffuse GFP signal from inside the vacuole suggests degradation of *Ca*Nce102-GFP (Fig. 8C to E).

Our data show that Nce102 behaves analogously in a laboratory S. cerevisiae strain and a C. albicans strain derived from a clinical isolate, pointing to the conservation of the sensing principle. The reported changes of Nce102 localization and level are more extensive in the pathogen, corresponding to its need to be able to react rapidly to changes in the environment caused by the immune system response of the host organism.

## DISCUSSION

Here, we bring new important insights into the function of the plasma membrane microdomain protein Nce102, specifically in the sensing of biophysical properties and lipid composition of the plasma membrane. We show that the Nce102 protein level and localization reflect the amount of cellular sphingolipids being produced during increased sphingolipid demand and internalized in their excess. At the same time, cells lacking the *NCE102* gene have altered sphingolipid landscape under both basal and stress conditions. We found that Nce102 behaves analogously in the human fungal pathogen Candida albicans, pointing to the potential clinical relevance of the described principle.

We report that Nce102 redistribution out of the MCC and into the surrounding plasma membrane following myriocin-induced inhibition of sphingolipid biosynthesis requires an active cell cycle (Fig. 4). The available data indicate that the decrease of sphingolipids in the plasma membrane of mother cells is probably due to their endocytosis and rerouting to the growing bud via inheritance of organelles. While we show that endocytosis remains active in the time frame of our experiments, sphingolipids could also be internalized by nonvesicular transport at membrane contact sites between the plasma membrane and either endoplasmic reticulum and/or Golgi. This mechanism predicts the existence of a phospholipase C that would convert the complex sphingolipids in the outer leaflet of the plasma membrane into ceramide, which has been shown to readily flip-flop in large unilamellar vesicles (70). To what extent this function could be accomplished by Isc1, an inositol phosphosphingolipid phospholipase C localized to the endoplasmic reticulum and mitochondria, is unknown. To date, the plasma membrane activity of Isc1 has not been reported (71, 72). In turn, tricalbins might be involved in the actual transport from the plasma membrane to the ER/Golgi since they are known tethers of cortical ER and plasma membrane and have a ceramide-binding domain (73, 74).

### The distribution of Nce102 reflects local demand for sphingolipids.

In our microscopy images of exponentially growing cultures, Nce102-GFP distributed homogeneously within the plasma membrane of growing buds even in the absence of sphingolipid biosynthesis inhibitors (Fig. 1A and Fig. 3A and B; see Fig. S8 in the supplemental material). As the buds became larger, Nce102-GFP patches started to form, initially predominantly in areas close to the bud neck, where the membrane was already matured. This finding is consistent with a previous report showing that eisosomes (the cytoplasmic hemitubules directly interacting with the MCC) are formed only after the growing bud reaches a critical surface area of ~20 μm^2^. As the bud grows further, more eisosomes are gradually formed, starting at the bud neck and progressing toward the bud tip (75). Importantly, the MCC protein Sur7 in C. albicans behaves analogously (69). The newly formed plasma membrane in the growing bud is expanding rapidly and presents an area with high sphingolipid demand, analogous to the cellular plasma membrane under conditions of inhibition of sphingolipid biosynthesis. Nce102 is distributed homogeneously in both cases. We, therefore, propose that Nce102 is a local signal of increased sphingolipid demand when localized outside the MCC in a particular plasma membrane area. We have shown previously an analogous regulation of activity by sequestration into the MCC/eisosome for the exoribonuclease Xrn1 that is inactivated by binding to the eisosome in postdiauxic cultures (76). Several transporters behave similarly, accumulating in the MCC when their substrates are unavailable (5, 77–79).

According to the current model, migration of Nce102 out of MCC triggers eisosome disassembly and the release of TORC2 activators Slm1,2 (27–29). It has been proposed that Nce102 itself could, together with another integral plasma membrane protein Sng1, interact physically with TORC2. These proteins were suggested to create a scaffold enhancing the interaction of Ypk1 with TORC2, increasing the effectiveness of sphingolipid biosynthesis activation via the TORC2 → Ypk1 → SPT (serine palmitoyltransferase) pathway under such circumstances (66). Physical interaction of Nce102 with Tor1 and Tor2 has indeed been reported previously (80). The increase in the Nce102 level in the plasma membrane that we report here should therefore lead to increased TORC2 activation and hence higher overall activity of the sphingolipid biosynthetic pathway. Consistently, overexpression of *NCE102* has been reported to prevent eisosome disassembly in response to myriocin treatment (6). We also show that creating a state of surplus availability of sphingolipid precursors leads to the internalization of Nce102 into the vacuolar membrane (Fig. 3B), which could prevent excessive activation of TORC2. We have shown recently (40) (also apparent in Fig. 6A) that in postdiauxic cells when the cellular sphingolipid levels are high, a part of the Nce102 population localizes to the ergosterol-rich domains of the vacuole. This part might represent a pool of Nce102 “ready to be put into action” should the need arise for a fast increase in sphingolipid biosynthesis. Whether the Nce102 localized to the vacuolar membrane can be redirected back to the plasma membrane under such conditions remains to be verified.

### Mechanisms governing Nce102 sequestration in the MCC in response to sphingolipid levels.

One question that remains to be answered is how Nce102 senses local sphingolipid levels. One option is that the Nce102 molecule changes conformation in response to a local drop in sphingolipid concentration. This option is favored by a previous study that analyzed the flexible membrane topology of the Nce102 protein (18) as well as by the fact that the vacuolar fraction of Nce102 occupies membrane microdomains of the curvature opposite to that of MCC (40). Analogous to the arginine importer Can1, conformational change in the Nce102 molecule could result in lower clustering of Nce102 in the curved membrane of the MCC microdomain (77). In the case of Can1 and other MCC transporters, the conformational change is triggered by substrate binding (8, 77). For Nce102, another mechanism needs to be considered, for example, a posttranslational protein modification. Since an intact C-terminal domain of Nce102 is essential for the protein’s accumulation in the MCC (18), this area appears to be a good candidate in this respect. In fact, two recent high-throughput studies have identified T162 and S171 in the cytosolic C-terminal tail of Nce102 as potential phosphorylation sites (81, 82). In the context of the results of this study, it is interesting to mention that it appears that the cyclin-dependent kinase Cdk1 might be involved in the phosphorylation of T162 in a cell cycle-dependent manner (83). The phosphorylation status of Nce102 could be regulated by long-chain bases, as is the case of the eisosome constituents Pil1 and Lsp1 (10). The changes in Nce102 phosphorylation in response to myriocin treatment are under study currently and will be the subject of another publication.

Here, we report that not only a lack but also a surplus of sphingolipid precursors cause Nce102 to migrate out of the MCC (Fig. 3). Incorporation of exogenous PHS (and complex sphingolipids newly synthesized from it) into the plasma membrane increases its sphingolipid amount, disrupting the established sphingolipid-to-ergosterol balance. This process leads to a decrease in plasma membrane fluidity that the cell needs to compensate. In such a situation, the MCC might serve as an ergosterol reservoir, providing the necessary interaction partners for the surplus sphingolipids. As Nce102 localizes preferentially into ergosterol-rich domains of membranes (4, 40, 84), this change in the lipid landscape could result in redistribution of Nce102 out of the MCC. Once outside, Nce102 is accessible to endocytic machinery and can be internalized to the vacuolar membrane under specific conditions, such as an excess of LCBs. Consistently, it has been shown that exogenous PHS induces actin-dependent endocytosis and degradation of the MCC-accumulating uracil permease Fur4, which requires the Rsp5 ubiquitin ligase (85, 86). In the case of Nce102, we show that the internalization is dependent on the multivesicular body endocytic pathway (Fig. 3), consistent with our previous report (40), and is not connected to degradation. Under basal conditions, Nce102 is distributed between the MCCs and the surrounding plasma membrane. Upon disturbance in the sphingolipid balance in either direction, Nce102 is released from the MCC, and its level in the plasma membrane is tuned accordingly, either by *de novo* synthesis or internalization.

### Functional interplay between Nce102 and additional enzymes of the sphingolipid biosynthesis pathway.

Our lipid analysis showed that the sphingolipid landscape is affected by the absence of *NCE102*. Besides the changes in the overall sphingolipid content, specific lipid classes were affected differentially by the absence of this proposed sphingolipid sensor. Interestingly, while the sphingolipid content of the *nce102* deletion mutant cells was generally decreased in the absence of stress, the levels of phytoceramides were elevated. This finding suggests the possibility of a functional interplay between Nce102 and either IPC synthase (Aur1-Kei1) or Isc1, a yeast homolog of mammalian sphingomyelinases (87), responsible for the conversion of complex sphingolipids back into ceramides. Furthermore, hydroxylated sphingolipid species were decreased in the *nce102* deletion mutant compared with the wild type in response to heat stress, suggesting that there might be a further possible functional interplay between Nce102 and Scs7, which mediates α-hydroxylation of PHS and DHS (88). It has been shown that while *scs7*Δ cells have unchanged sterol composition, the sphingolipid composition is very different from the wild type, resulting in a higher abundance of highly ordered sphingolipid-rich plasma membrane domains (64). These data suggest not only that Nce102 might be involved in the regulation of the overall rate of sphingolipid biosynthesis (via regulation of TORC2 activation, as discussed above), especially in response to heat stress, but also that it might be functionally connected to additional enzymes of the pathway. The resulting impact on the sphingolipid landscape is condition dependent and deserves to be studied in more detail.

In conclusion, our study provides important insights into the mechanisms by which the cell perceives the levels of sphingolipids in the plasma membrane and relays this information downstream. Specifically, we identified Nce102 as a local marker of increased sphingolipid demand within the plasma membrane. We further show that the amount of the protein fine-tunes sphingolipid synthesis, probably via interaction with TORC2 and/or specific enzymes along the actual sphingolipid biosynthesis pathway. Uncovering the details of these interactions will be the objective of further studies. While disruptions of the MCC microdomain rarely lead to a growth-related phenotype in laboratory strains of S. cerevisiae, they are detrimental to morphogenesis and invasive growth of C. albicans (89–91). Our study adds to the growing body of data indicating that the MCC/eisosome is a viable target in the treatment of fungal infections (92, 93).

## MATERIALS AND METHODS

### Strains and growth conditions.

S. cerevisiae and C. albicans strains used in this study are listed in Table 1. The Y786 strain was constructed by genomic tagging of *PIL1* with *mRFP* in the Y549 strain using the YIp128-*PIL1-mRFP* integrative plasmid as described previously (94). Yeast cultures were cultivated in synthetic complete medium (SC; 0.17% yeast nitrogen base without amino acids and ammonium sulfate, 0.5% ammonium sulfate, and 2% dextrose) supplemented with required amino acids. In the case of C. albicans, 80 mg/L uridine was used instead of uracil. S. cerevisiae cultures were grown in an incubator on an orbital shaker at 220 rpm at either 28°C (microscopy) or 30°C (Western blot) and C. albicans in a tube roller at 37°C. Overnight cultures were diluted to an optical density at 600 nm (OD_600_) of 0.2 in fresh medium to start the main cultures which were then incubated for a desired time.

**TABLE 1:** Strains used in the study

Strain by species	Genotype	Source
S. cerevisiae		
BY4742	*MATα his3Δ1 leu2Δ0 lys2Δ0 ura3Δ0*	JBK lab stock; Euroscarf
*nce102*Δ	BY4742 *nce102*Δ*::kanMX*	Provided by Bruce Futcher, Stony Brook University, NY
Y172	BY4742 *LSP1-GFP::LEU2*	This work
Y193	BY4742 *SUR7-GFP::URA3*	4
Y240	BY4742 *NCE102-GFP::URA3*	40
Y549	BY4742 *SLM1-GFP::HIS3*	Provided by V. Strádalová, Université de Fribourg, Switzerland
Y786	Y549 *PIL1-mRFP::LEU2*	This work
Y1009	BY4742 *vps4*Δ*::kanMX NCE102-GFP::URA3*	40
Y1039	BY4742 *TRP1::ss-dsRed-HDEL::URA3 NCE102-GFP::LEU2*	40
C. albicans		
BWP17	*ura3Δ::λimm434/ura3Δ::λimm434 his1::hisG/his1::hisG arg4::hisG/arg4::hisG*	105
YHXW15	BWP17 *NCE102-GFPγ::URA3*	90
YJA15	BWP17 *SUR7-GFPγ::URA3*	69

### Stress treatment.

To study the effects of acute stress, cultures were grown as described above for 6 h and exposed to stress for 2 h. For chemically induced stress, ethanol (96%), fluconazole (1 or 2 mg/mL ethanol stock), myriocin (2 mg/mL methanol stock), cycloheximide (10 mg/mL water stock), phytosphingosine (PHS; 5 mg/mL ethanol stock), dihydrosphingosine (DHS; 5 mg/mL methanol stock), hydroxyurea (2 M stock in SC, 28°C, freshly prepared), and FM4-64 (16 mM DMSO stock) were added to the desired concentrations. All chemicals except ethanol (Penta) were purchased from Merck. Oxygen limitation was induced by growing a large volume of cell culture aerobically and then replacing 33 mL into a 50-mL Falcon tube that was closed tightly and sealed with parafilm to prevent air exchange with the surroundings. For heat stress, S. cerevisiae cells were shifted from 28 to 37°C; C. albicans was shifted from 37 to 30°C. Chronic stress was induced by the same agents applied upon inoculation of main cultures. They were grown for 6, 24, or 48 h and processed for respective experiments as described below.

### Confocal fluorescence microscopy of live cells.

Yeast cultures were grown as described above and concentrated by brief centrifugation. A total of 1 μL of cell suspension was immobilized on a 0.17-mm cover glass by a thin film of 1% agarose prepared in 50 mM potassium phosphate buffer (pH 6.3). For time-lapse experiments of exponential cultures, the 1% agarose was prepared in fresh SC medium to supplement nutrients. S. cerevisiae cells were imaged using Zeiss LSM 880 and Olympus BX61WI scanning confocal microscopes. For C. albicans cells, 9- to 11-layered z-stacks were made using a Zeiss Axio Observer Z1/7 wide-field fluorescence microscope. All microscopes were equipped with a 100× PlanApochromat oil immersion objective (numerical aperture [NA], 1.4). GFP and FM4-64 were excited using the 488- and 514-nm lines of the Argon laser, respectively. mCherry/dsRed/mRFP were excited using the 561-nm DPSS laser. C. albicans images were drift corrected and deconvolved in the proprietary Zeiss ZEN software, Black edition.

### Image analysis.

Image processing and analysis were performed in Fiji (ImageJ 1.53c) using custom-written macros, available online at https://github.com/jakubzahumensky/Nce102_SL_paper (this work) and https://github.com/jakubzahumensky/Isc1_paper (95), and cell segmentation masks were made using Cellpose software (96). In brief, fluorescence channels to be quantified were exported from the raw microscopy images into TIFF format and used to make cell segmentation masks in Cellpose. The segmentation parameters were set to make the mask edges intersect plasma membrane patches in their middles. Incompletely imaged cells were removed automatically at this step. The segmentation masks were converted into regions of interest (ROIs) in Fiji, fitted with ellipses as approximations of cells, and curated manually to remove or adjust incorrectly created ROIs. For each cell, multiple parameters were quantified, as follows: mean and integrated fluorescence signal intensity (whole cell/plasma membrane/cell interior), cell cross-section area, and number of high-intensity patches in the plasma membrane. The mean values of parameters of interest were calculated from all cells within each biological replicate. From them, the final mean and standard deviation were calculated and plotted. For the Slm1-GFP/Pil1-mRFP colocalization analysis, the fluorescence channels were shift-corrected, and the Pearson’s correlation coefficient was calculated using ZEN Software, Blue edition.

### Western blot.

For the analysis of changes in *Sc*Nce102 protein abundance following inhibition of sphingolipid biosynthesis, exponential wild-type BY4742 cultures were processed as described previously (40) with the following modifications: pellets obtained by harvesting were frozen immediately in liquid nitrogen and kept at −80°C overnight, thawed on ice, and resuspended in TNE buffer (50 mM tris-HCl, 150 mM NaCl, 5 mM ethylenediaminetetraacetic acid in H_2_O, [pH 7.5]) containing protease inhibitors. Next, 5× Laemmli sample buffer was added to homogenates diluted to the desired concentration, and samples were boiled at 95°C for 5 min. Primary antibodies were as follows: Nce102—αNce102 rabbit polyclonal (1:1,000) (18); and αTubulin rat monoclonal (1:10,000, ab6160; Abcam; loading control). Horseradish peroxidase (HRP)-conjugated secondary antibodies were Nce102—anti-rabbit; and tubulin—anti-rat (goat, 1:10,000; Jackson ImmunoResearch). HRP chemiluminescence was monitored with a c400 (Azure Biosystems) detection system and analyzed using Image Studio Lite v. 5.2. Statistical analysis was performed in GraphPad Prism 9 software.

To monitor changes in Nce102 abundance following chronic stress, yeast cultures (S. cerevisiae, BY4742; C. albicans, YHXW15) were grown for 48 h as described above, harvested by centrifugation, and resuspended in 300 μL 1× sample buffer (2% SDS, 10% glycerol, and 125 mM Tris-HCl [pH 6.8]). Cells were lysed using zirconia beads by 4 rounds of beating. The protein amount was measured using the Pierce bicinchoninic acid (BCA) protein assay (Thermo Fisher Scientific), and samples were diluted to the desired concentration. Bromophenol blue (0.002%) and 2-beta-mercaptoethanol (2%) were added and samples boiled at 95°C for 5 min. Proteins were resolved by SDS-PAGE and transferred to a nitrocellulose membrane using a semidry transfer apparatus. Following 60-min blocking in 5% skim milk in Tris-buffered saline with Tween 20 (TBST) buffer (20 mM Tris‐HCl [pH 7.6], 150 mM NaCl, and 0.05% Tween20), the membranes were incubated overnight at 4°C in 0.05% NaN_3_ containing 1% skim milk in TBST buffer with respective primary antibodies as follows: *Sc*Nce102—αNce102 rabbit polyclonal (1:1,000) (18); and *Ca*Nce102-GFP—αGFP (1:1,000; mouse; Living Colors A.v. monoclonal antibody [JL-8]). The membranes were washed with TBST and incubated at room temperature in the dark for 1 h, in 1% skim milk in TBST buffer with respective secondary antibodies, as follows: αRabbit (1:20,000; goat; Li-Cor; IRDye 800CW goat anti-rabbit IgG [H+L]) and αMouse (1:20,000; goat; Li-Cor; IRDye 800CW goat anti-mouse IgG [H+L]). For loading normalization, SDS-PAGE as described above was performed in parallel. The gels were stained in a Coomassie blue solution (0.1% Coomassie brilliant blue R-250, 40% methanol, and 10% glacial acetic acid) for 1 h and destained (40% methanol, 10% glacial acetic acid) overnight on a shaker. Both the membranes and Coomassie blue-stained gels were imaged using an Odyssey CLx infrared scanner and analyzed using Image Studio Lite v. 5.2 software (Li-Cor Biosciences).

### Lipid analysis.

S. cerevisiae BY4742 and BY4742 *nce102*Δ cultures were grown for 48 h as described, washed twice with distilled water, and harvested by centrifugation. The cells were counted manually using a Bürker chamber, and aliquots of 5.10^8^ cells total were made. The suspensions were pelleted, and C17-sphingolipids were added before cell lysis to be used as internal standards (97, 98). Mandala extraction was carried out as described previously (99), followed by Bligh and Dyer extraction (100). A third of each sample obtained from the Bligh and Dyer extraction was reserved for inorganic phosphate (P_i_) determination. The remaining two-thirds of the organic phase was transferred to a new tube and submitted to alkaline hydrolysis of phospholipids (101). The organic phase was dried and used for lipid chromatography-mass spectrometry (LC-MS) analysis as described previously in detail (98). The sphingolipid levels reported by the Mass-Spec facility are calculated from raw spectral data using internal standards (Table S1, sheet “RAW”). They were normalized by P_i_ abundance in each sample. M(IP)_2_Cs were not detected in our assay. The used sphingolipid has been used previously elsewhere (39, 102–104).

For the heat map in Fig. 7C, the amounts of sphingolipids were normalized to the respective control (*nce102*Δ control to wild-type control) and converted to a log_2_ scale in GraphPad Prism 9. For bar charts in Fig. S7, lipid amounts were pooled to indicate lipid classes. The correlation analysis (Fig. 7D) was performed using sphingolipid amounts of the wild-type strain only (Table S1) and Nce102 amounts obtained by Western blot analysis (Fig. 6E) for respective stress conditions. Only respective amounts were considered to calculate the correlation coefficients.

### Statistical analysis.

Statistical analysis was performed in GraphPad Prism 9 software. Statistical significance was determined by either *t* test or one-way or two-way analysis of variance (ANOVA), depending on the experimental setup. Data distribution was assumed to be normal (except for the myriocin effect on the Nce102 patch number) but was not tested formally.

### Data availability.

All data supporting the findings reported here are available within the manuscript and its associated files. No data were deposited in external repositories. Custom-written Fiji (ImageJ) macros used for microscopy image analysis are available on GitHub, including sample data (https://github.com/jakubzahumensky/Nce102_SL_paper).
