# Supplementary material for: Microdomain Protein Nce102 Is a Local Sensor of Plasma Membrane Sphingolipid Balance
Source: Microbiol Spectr. 2022 Jun 27;10(4):e01961-22. doi: 10.1128/spectrum.01961-22 (PMC9431316; doi:10.1128/spectrum.01961-22)
Supplement: Supplemental file 1 — Fig. S1-S8. Download spectrum.01961-22-s0001.pdf, PDF file, 1.7 MB [file spectrum.01961-22-s0001.pdf]

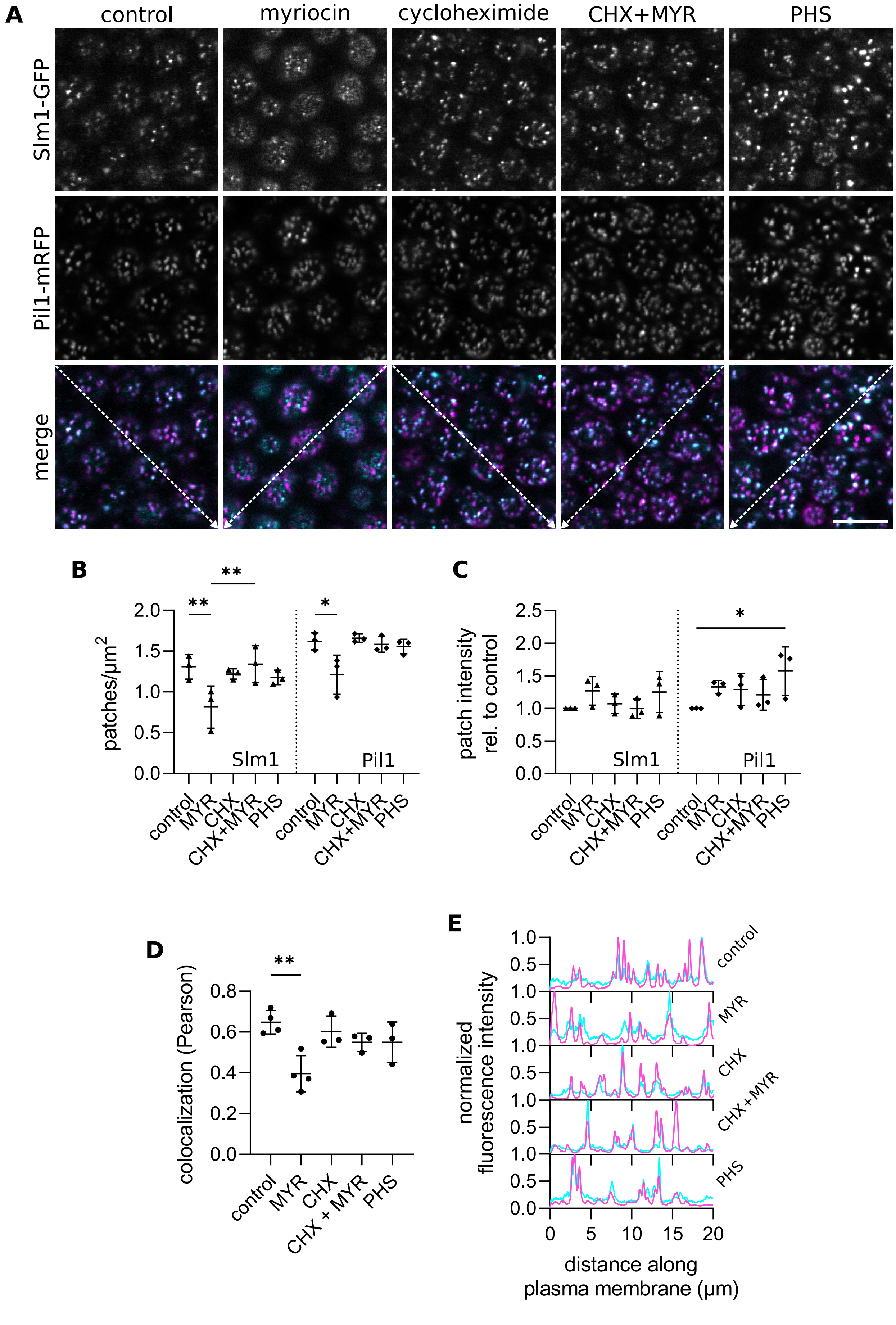

Fig S1 – Inhibition of sphingolipid biosynthesis induces release of Slm1 from the eisosome – tangential focal planes.

A Confocal microscopy images of *S. cerevisiae* cells expressing *SLM1-GFP* together with the core eisosome protein *PIL1-mRFP* cultivated for 6 hours and exposed to indicated chemicals for 2 hours. Scale bars: 5 μm. Dashed lines with arrows indicate cells and direction of line intensity plots displayed in (E).

B–D Quantification of the density of Slm1-GFP and Pil1-mRFP patches (B), mean patch intensity (C) and Pearson's colocalization coefficient of Slm1-GFP and Pil1-mRFP (D) in cultures treated as in (A). Mean ± SD from 3-5 biological replicates (Slm1-GFP – triangles, Pil1-mRFP – diamonds, colocalization – dots; 150–200 cells in each condition). * – $P \leq 0.05$; ** – $P \leq 0.01$. One-way ANOVA.

E Normalized line intensity plots of Slm1-GFP (cyan) and Pil1-mRFP (magenta) across the microscopy images as indicated in (A).

Myriocin (MYR) – 10 μM, cycloheximide (CHX) – 100 μg/ml, phytosphingosine (PHS) – 20 μM.

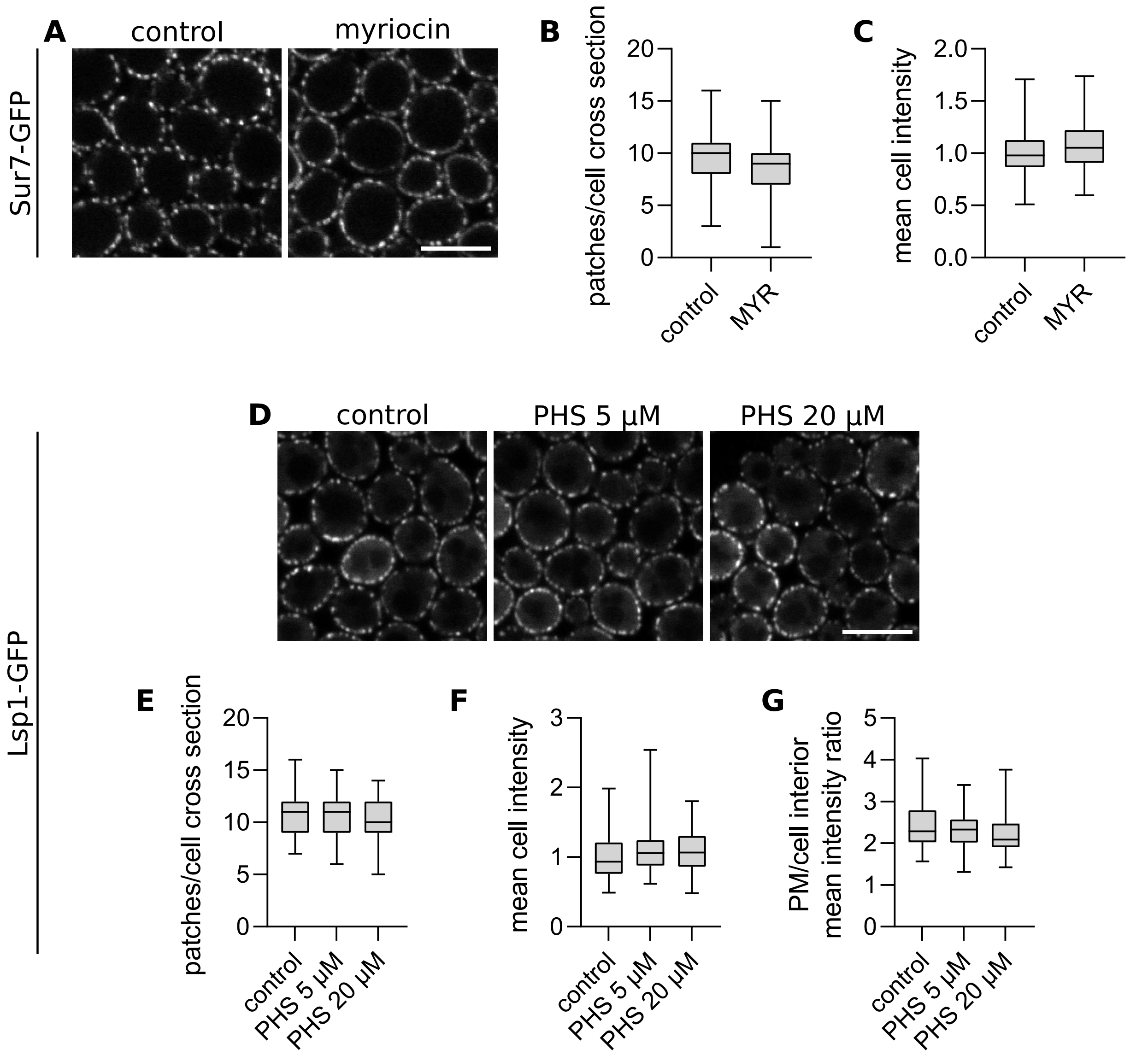

Fig S2 – Changes in localization in response to sphingolipid amount modulation are unique to Nce102.

A Confocal microscopy images of S. cerevisiae cells expressing *SUR7-GFP* cultivated for 6 hours and exposed to 10 µM myriocin for 2 hours. Scale bars: 5 µm.

B, C Quantification of the number of Sur7-GFP patches per cell cross-section (B) and mean cell GFP intensity (C) in cultures treated as in (A). Box plot with median and range (min to max) indicated. One biological replicate (control – 230 cells, myriocin – 330 cells).

D Confocal microscopy images of *S. cerevisiae* cells expressing *LSP1-GFP* cultivated for 6 hours and exposed to PHS at indicated concentrations for 2 hours. Scale bars: 5 µm.

E–G Quantification of the number of Lsp1-GFP patches per cell cross-section (E) and mean cell GFP intensity (F), and the ratio of mean GFP fluorescence intensity in the plasma membrane (PM) and in the cell interior (G) in cell cultures treated as in (D). Box plot with median and range (min to max) indicated. One biological replicate (115–130 cells in each condition).

MYR – myriocin, PHS – phytosphingosine

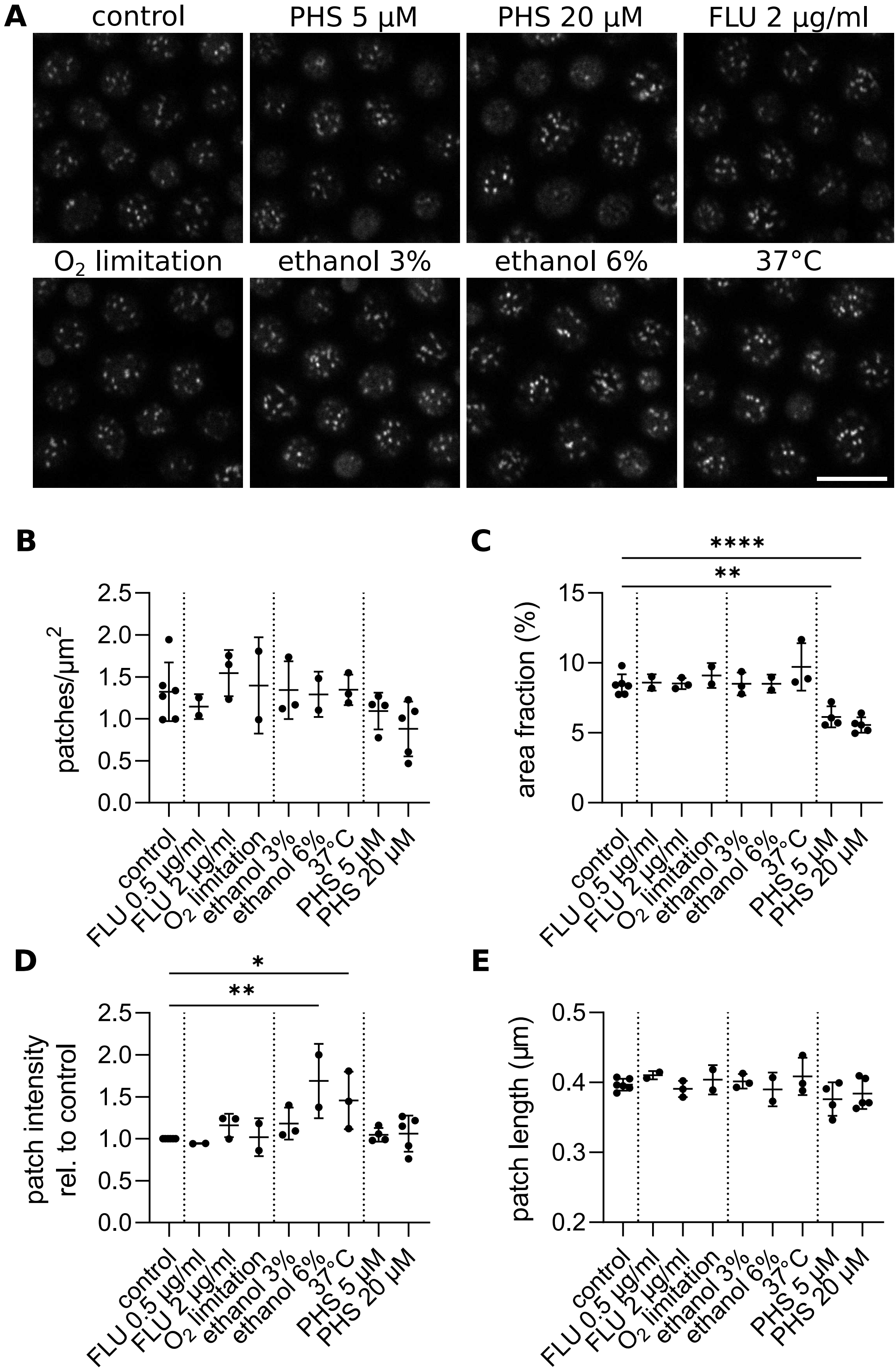

Fig S3 – Nce102 localization and abundance change in response to increasing plasma membrane fluidity and sphingolipid levels – tangential focal planes.

A Confocal microscopy images of *S. cerevisiae* cells expressing *NCE102-GFP* cultivated for 6 hours and exposed to indicated stress conditions for 2 hours. Scale bar: 5 µm.

B–E Quantification of the density of (B), plasma membrane fraction taken up by (C), mean intensity of (D) and length of (E) Nce102-GFP patches in cultures treated as in (A). Mean ± SD from 2-5 biological replicates (dots; 300–400 cells in each condition). * – P ≤ 0.05; ** – P ≤ 0.01; **** – P ≤ 0.0001. One-way ANOVA. No statistical difference was found between conditions in (B). Vertical dotted lines separate groups of stress conditions – left to right: control, ergosterol biosynthesis inhibition, increase of membrane fluidity.

PHS – phytosphingosine, FLU – fluconazole

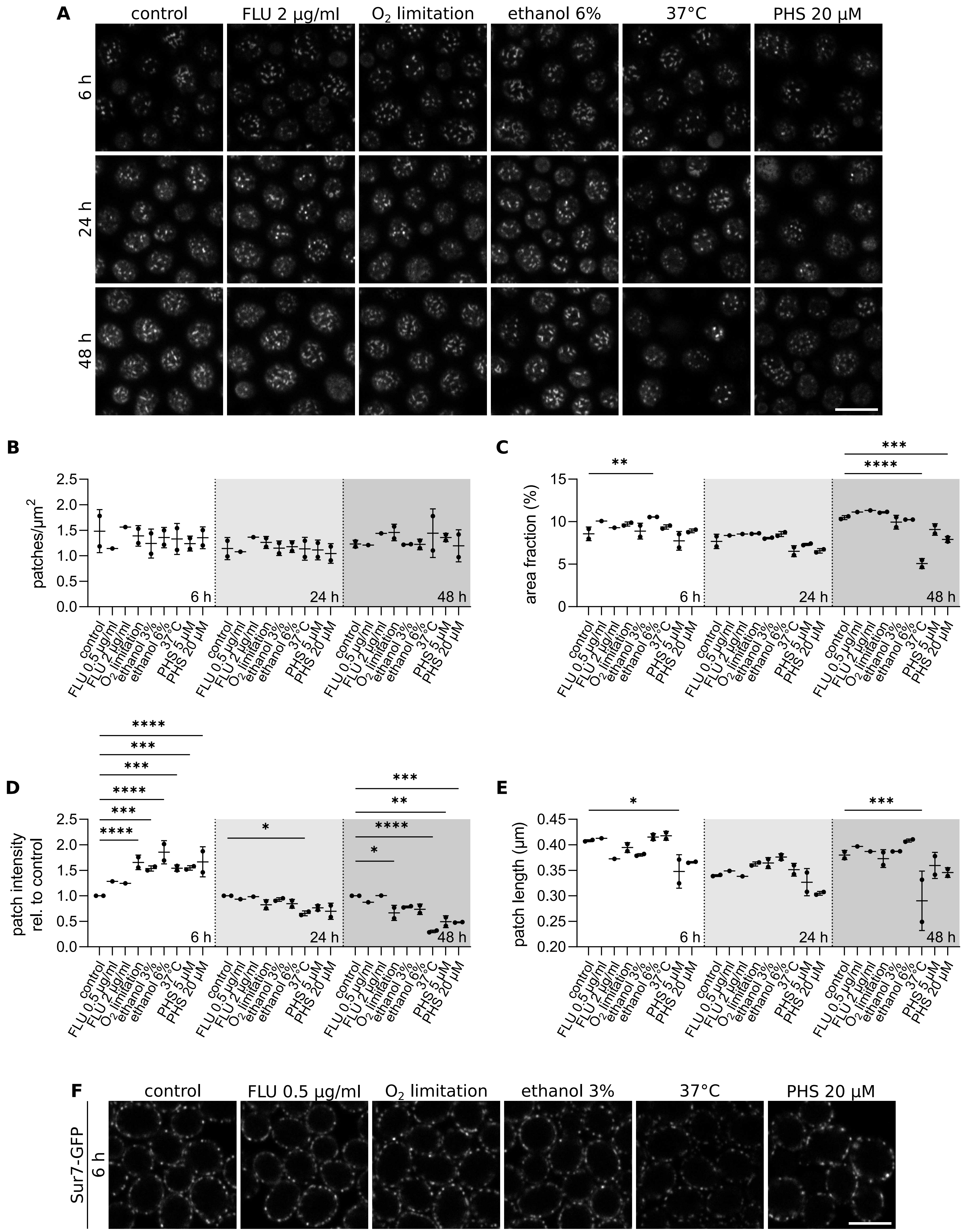

Fig S4 - Nce102 response to chronic stress – tangential focal planes.
A Confocal microscopy images of *S. cerevisiae* cells expressing *NCE102-GFP* treated with indicated stress upon inoculation and cultivated for the indicated time. Scale bars: 5 µm.
B–E Quantification of the density of (B), plasma membrane fraction taken up by (C), mean intensity of (D) and length of (E) Nce102-GFP patches in cultures treated as in (A). Mean ± SD from 3 biological replicates (dots; 270–500 cells per condition; dead cells were excluded from analysis). * – P ≤ 0.05; ** – P ≤ 0.01; *** – P ≤ 0.001; **** – P ≤ 0.0001. One-way ANOVA. No statistical difference was found between conditions in (B). Vertical dotted lines separate cultivation times.
F Confocal microscopy images of *S. cerevisiae* cells expressing *SUR7-GFP* treated with indicated stress upon inoculation and cultivated for the indicated time. Scale bars: 5 µm.
FLU – fluconazole, PHS – phytosphingosine

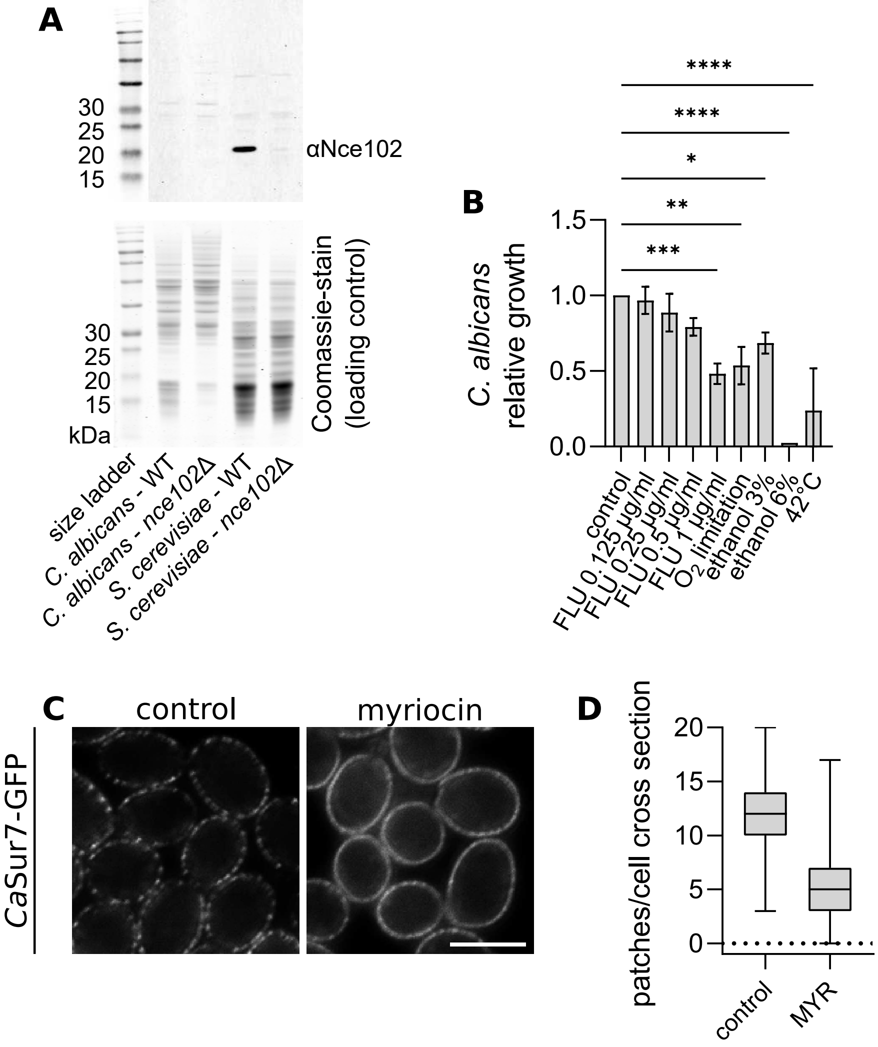

Fig S5 – Specificity of αNce102 antibody and the effect of chronic stress on the growth of *C. albicans*.
A *C. albicans* and *S. cerevisiae* wild type (WT) and *nce102*Δ cultures were grown for 24 hours and analyzed by western blot for αNce102 antibody specificity.
B *C. albicans* cultures were treated with indicated stress upon inoculation and cultivated for 48 hours. OD_{600} was measured and growth relative to control was calculated. FLU – fluconazole. 3 biological replicates. * – $P \leq 0.05$; ** – $P \leq 0.01$; *** – $P \leq 0.001$; **** – $P \leq 0.0001$. One-way ANOVA.
C Deconvolved wide-field fluorescence microscopy images of *C. albicans* cells expressing *CaSUR7-GFP* cultivated for 6 hours and exposed to myriocin for 2 hours. Scale bars: 5 µm.
D Quantification of the number of CaSur7-GFP patches per cell cross-section following 2-hour myriocin treatment. Box plots with median and range (min to max) indicated. One biological replicate (control – 1100 cells, myriocin – 980 cells).
MYR – myriocin

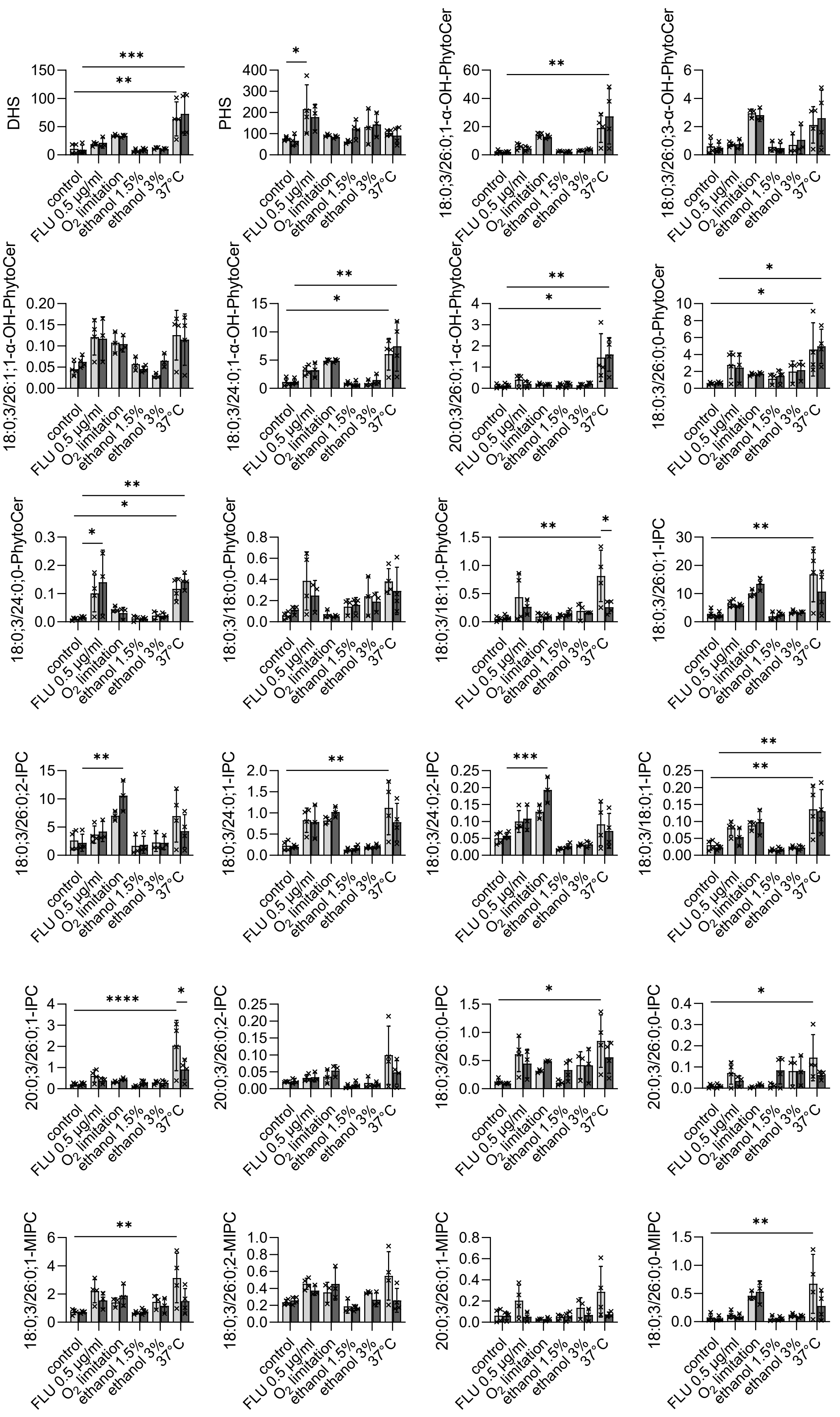

Fig S6 – Changes in the levels of individual sphingolipids in response to chronic stress.

S. cerevisiae wild type (WT) and *nce102*Δ cultures were treated with indicated stress conditions upon inoculation and cultivated for 48 hours (same as in Fig 6). All lipid amounts are in (pmol lipid/pmol Pi). Bars indicate mean ± SD calculated from 3-4 biological replicates (crosses). Light gray – wild type, dark gray – *nce102*Δ. * – P ≤ 0.05, ** – P ≤ 0.01, *** – P ≤ 0.001, **** – P ≤ 0.0001. One-way ANOVA.

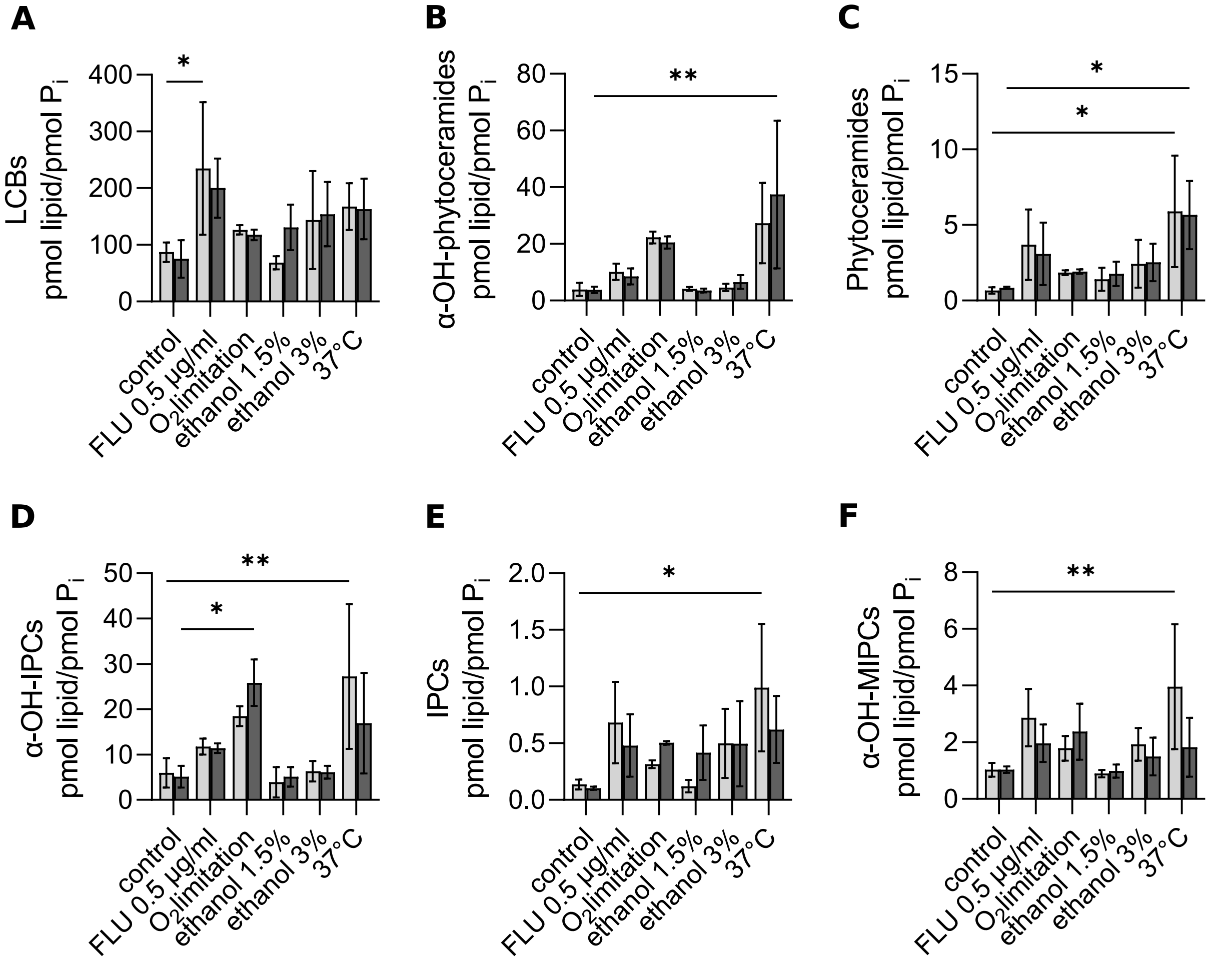

Fig S7 – Changes in the levels of sphingolipid classes in response to chronic stress.

S. cerevisiae wild type (WT) and *nce102Δ* cultures were treated with indicated stress conditions upon inoculation and cultivated for 48 hours (same as in Fig 6). Lipids from indicated classes were pooled and differences in their amounts analyzed. Bars indicate mean ± SD calculated from 3-4 biological replicates. Light gray – wild type, dark gray – nce102Δ. Bars represent means ± SD. * – $P \leq 0.05$, ** – $P \leq 0.01$. One-way ANOVA.

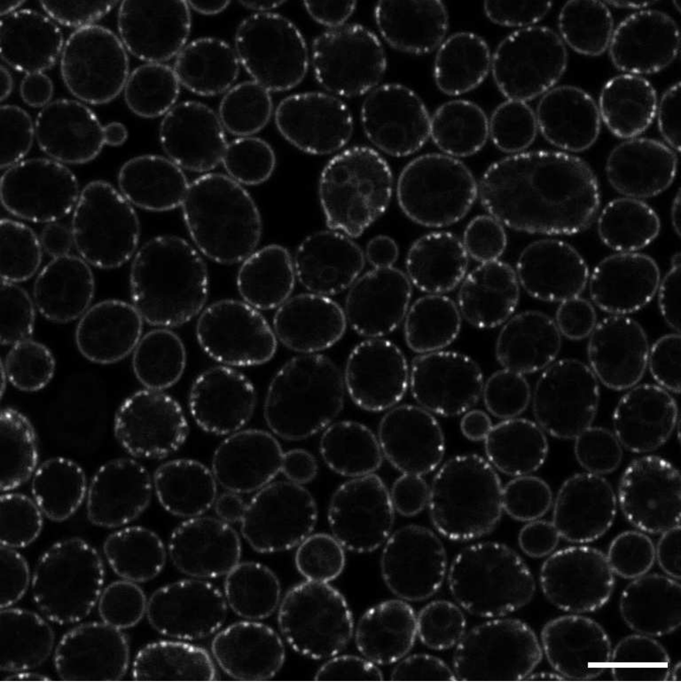

Fig S8 – Nce102-GFP distribution in growing *S. cerevisiae* buds.
Confocal microscopy image of *S. cerevisiae* cells expressing *NCE102-GFP* cultivated for 8 hours (corresponding to control sample in Fig 1A). Scale bar: 5 μm.
