# Supplementary material for: Microdomain Protein Nce102 Is a Local Sensor of Plasma Membrane Sphingolipid Balance
Source: Microbiol Spectr. 2022 Jun 27;10(4):e01961-22. doi: 10.1128/spectrum.01961-22 (PMC9431316; doi:10.1128/spectrum.01961-22)
Supplement: Supplemental file 2 — Legends for Movie S1 and Table S1. Download spectrum.01961-22-s0002.pdf, PDF file, 0.03 MB [file spectrum.01961-22-s0002.pdf]

Movie S1 – Time-lapse of the change of Nce102-GFP distribution in response to myriocin treatment. *S. cerevisiae* cells expressing *NCE102-GFP* were cultivated for 6 hours, treated with 10 µM myriocin and imaged in a time-lapse manner. Scale bar: 5 µm. Time indicated in the Movie.

Table S1 – Mass spectrometry lipid analysis data (source data for Fig 7, S6 and S7)
Lipid amounts corrected with internal standards were delivered by the lipidomics facility (A), normalized by inorganic phosphate amount (B) and then normalized either to respective control mean (C) or to wild type control mean (D).
